# Pharmacological characterisation of the highly Na_V_1.7 selective spider venom peptide Pn3a

**DOI:** 10.1038/srep40883

**Published:** 2017-01-20

**Authors:** Jennifer R. Deuis, Zoltan Dekan, Joshua S. Wingerd, Jennifer J. Smith, Nehan R. Munasinghe, Rebecca F. Bhola, Wendy L. Imlach, Volker Herzig, David A. Armstrong, K. Johan Rosengren, Frank Bosmans, Stephen G. Waxman, Sulayman D. Dib-Hajj, Pierre Escoubas, Michael S. Minett, Macdonald J. Christie, Glenn F. King, Paul F. Alewood, Richard J. Lewis, John N. Wood, Irina Vetter

**Affiliations:** 1 IMB Centre for Pain Research, Institute for Molecular Bioscience, 306 Carmody Rd (Building 80), The University of Queensland, St Lucia, Queensland, 4072, Australia; 2 Discipline of Pharmacology, School of Medical Sciences, The University of Sydney, Sydney, New South Wales, 2006, Australia; 3 School of Biomedical Sciences, The University of Queensland, St Lucia, Queensland, 4072, Australia; 4 Department of Physiology & Solomon H. Snyder Department of Neuroscience, Johns Hopkins University, School of Medicine, Baltimore, MD 21205, USA; 5 Department of Neurology and Center for Neuroscience and Regeneration Research, Yale University School of Medicine, New Haven, Connecticut 06510, Rehabilitation Research Center, Veterans Administration Connecticut Healthcare System, West Haven, Connecticut 06516, USA; 6 Venomtech, Sophie-Antipolis, 06560, Valbonne, France; 7 Molecular Nociception Group, Wolfson Institute for Biomedical Research, University College London, London WC1E 6BT, UK; 8 School of Pharmacy, The University of Queensland, Pharmacy Australia Centre of Excellence, 20 Cornwall St, Woolloongabba, Queensland, 4102, Australia

## Abstract

Human genetic studies have implicated the voltage-gated sodium channel Na_V_1.7 as a therapeutic target for the treatment of pain. A novel peptide, μ-theraphotoxin-Pn3a, isolated from venom of the tarantula *Pamphobeteus nigricolor,* potently inhibits Na_V_1.7 (IC_50_ 0.9 nM) with at least 40–1000-fold selectivity over all other Na_V_ subtypes. Despite on-target activity in small-diameter dorsal root ganglia, spinal slices, and in a mouse model of pain induced by Na_V_1.7 activation, Pn3a alone displayed no analgesic activity in formalin-, carrageenan- or FCA-induced pain in rodents when administered systemically. A broad lack of analgesic activity was also found for the selective Na_V_1.7 inhibitors PF-04856264 and phlotoxin 1. However, when administered with subtherapeutic doses of opioids or the enkephalinase inhibitor thiorphan, these subtype-selective Na_V_1.7 inhibitors produced profound analgesia. Our results suggest that in these inflammatory models, acute administration of peripherally restricted Na_V_1.7 inhibitors can only produce analgesia when administered in combination with an opioid.

The pore-forming subunits of voltage-gated sodium channels (Na_V_) are integral membrane proteins that allow influx of sodium ions, which is essential for action potential generation and propagation in electrically excitable cells. They are comprised of four homologous but non-identical domains (DI–IV), each consisting of six α-helical transmembrane segments (S1–S6) connected by multiple intracellular and extracellular loops^1^,^2^. The channels are composed of two functionally distinct components: the S1–S4 transmembrane segments form the voltage-sensing domain that undergoes a conformational change in response to changes in membrane potential in order to initiate channel opening, while the S5–S6 segments form the pore region that controls ion selectivity and allows sodium influx. Mammals express nine Na_V_ channel subtypes (Na_V_1.1–1.9) that have distinct expression profiles and biophysical and pharmacological characteristics, with compelling genetic evidence linking Na_V_1.7 to pain^1^.

Loss-of-function mutations in *SCN9A*, the gene encoding Na_V_1.7, have been identified as a cause of congenital insensitivity to pain (CIP), a rare condition characterized by the inability to sense pain in individuals that are otherwise normal aside from anosmia (absence of sense of smell)^3^,^4^. Gain-of-function mutations of *SCN9A* are the cause of two hereditary pain disorders, inherited erythromelalgia (IEM) and paroxysmal extreme pain disorder (PEPD)^5^,^6^,^7^. Both disorders are associated with redness, swelling and burning pain, limited to the extremities in IEM and to the rectal, ocular and mandibular areas in PEPD. Therefore, pharmacological inhibition of Na_V_1.7 appears to be a promising therapeutic strategy for the treatment of pain. Developing analgesics with Na_V_1.7 selectivity is essential, as activity at the skeletal muscle isoform Na_V_1.4, the cardiac isoform Na_V_1.5, and the neuronal isoforms Na_V_1.1, Na_V_1.2 and Na_V_1.6 are likely to cause dose-limiting adverse effects^8^,^9^,^10^.

However, efforts to develop selective small molecule inhibitors have been hampered due to the high sequence identity (>50%) between Na_V_ subtypes, particularly in the pore forming segments (S5–S6) where local anaesthetics bind. Although some small molecule inhibitors of Na_V_1.7 that target the voltage-sensing domain have been described, the *in vivo* efficacy of these compounds in inflammatory and neuropathic pain animal models has not been reported^11^,^12^. We therefore know surprisingly little about the therapeutic potential of highly selective Na_V_1.7 inhibitors.

Spiders have evolved pharmacologically complex venoms dominated by disulfide-rich peptides, many of which act at ion channels to rapidly immobilise prey or deter predators^13^,^14^. Insect and vertebrate Na_V_ channel share 55–60% sequence identity and consequently many spider venom-derived peptides act on mammalian Na_V_ channels^15^,^16^,^17^. These venom peptides typically bind to the less conserved voltage-sensing domains, and hence they often achieve much better subtype selectivity than small molecules that bind to the pore region of the channel^14^,^18^.

Here, we report the isolation and characterization of μ-TRTX-Pn3a, a peptide isolated from venom of the South American tarantula *Pamphobeteus nigricolor*, and show that it has exquisite selectivity for Na_V_1.7. Using Pn3a, as well as the small molecule Na_V_1.7 inhibitor PF-04856264 and the spider venom peptide phlotoxin 1^11^,^19^,^20^, we assessed the therapeutic potential of selective Na_V_1.7 inhibition in multiple *in vivo* rodent models of pain. Surprisingly, despite on-target activity *in vivo*, these selective Na_V_1.7 inhibitors lacked analgesic activity in formalin-, carrageenan- and Freund’s complete adjuvant-induced pain models when administered alone. However, supporting the recently described crucial contribution of opioids to the Na_V_1.7 loss-of-function pain-free phenotype^21^, co-administration with a sub-effective dose of the opioids oxycodone or buprenorphine or the enkephalinase inhibitor thiorphan yielded analgesia, while co-administration with gabapentin had no effect. These results highlight the lack of broad analgesic efficacy in rodent models of pain with these peripherally restricted selective Na_V_1.7 inhibitors and confirm the proposed analgesic synergy between selective Na_V_1.7 inhibitors and opioids suggested by a recent study^21^.

## Results

### Isolation of the novel spider venom peptide μ-TRTX-Pn3a from *Pamphobeteus nigricolor*

**Figure 1:**
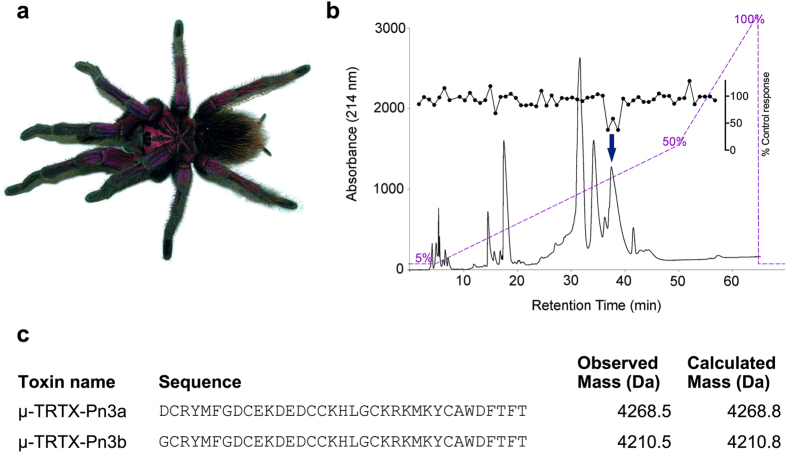
Isolation of the novel spider peptide μ-TRTX-Pn3a from the venom of *Pamphobeteus nigricolor.* (**a**) Photo of the male *Pamphobeteus nigricolor* specimen from which crude venom was obtained. (**b**) Chromatogram resulting from fractionation of the crude venom using RP-HPLC (purple dashed line indicates acetonitrile gradient). Corresponding activity of each fraction to inhibit veratridine-induced Na_V_1.3 responses is shown above (black circles). The arrow indicates the active peak. (**c**) Sequences of μ-TRTX-Pn3a and μ-TRTX-Pn3b identified by N-terminal sequencing.

Crude venom isolated from *Pamphobeteus nigricolor* (Fig. 1a) partially inhibited veratridine-induced membrane potential changes in HEK293 cells stably expressing rNa_V_1.3, with activity-guided fractionation isolating this activity to a single peak with a retention time of 38 min (Fig. 1b). Matrix assisted laser desorption/ionization time-of-flight mas spectrometry (MALDI-TOF MS) of this fraction revealed two dominant masses corresponding to monoisotopic masses of 4210.5 and 4268.5 Da. N-terminal sequencing identified two novel 35-residue sequences differing only by the N-terminal amino acid (Fig. 1c) that we named μ-TRTX-Pn3a (hereafter Pn3a) and μ-TRTX-Pn3b based upon the revised nomenclature recently proposed for spider-venom peptides^22^. We subsequently chose to investigate Pn3a, as this was the major sequence identified by the N-terminal sequencing. Chemical synthesis of Pn3a produced the correctly folded product, as native and synthetic Pn3a co-eluted when assessed using analytical HPLC (data not shown). Synthetic Pn3a was used for all further experiments.

### Pn3a is a potent and selective inhibitor of Na_V_1.7

**Figure 2:**
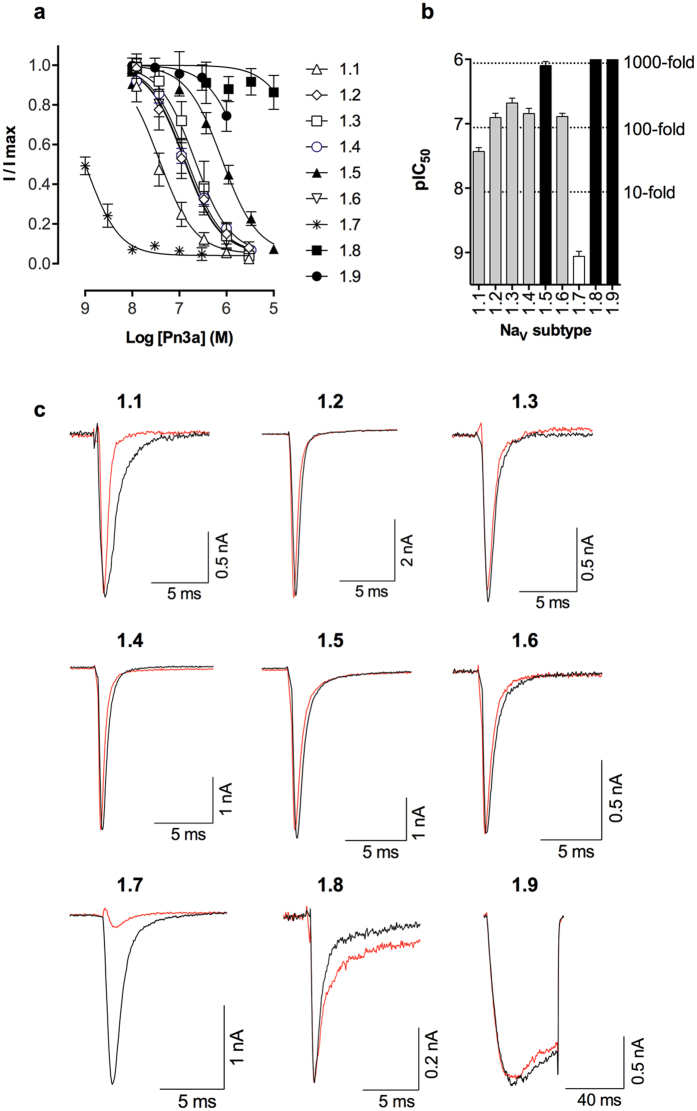
Selectivity of μ-TRTX-Pn3a for hNa_V_1.1-1.9 channels. (**a**) Concentration-response curves and (**b**) comparative potency of Pn3a at hNa_V_1.1-1.9 assessed by whole-cell patch-clamp experiments. Pn3a most potently inhibited Na_V_1.7, with 40-fold selectivity over hNa_V_1.1, 100-fold selectivity over hNa_V_1.2, 1.3, 1.4 and 1.6, and 900-fold selectivity over Na_V_1.5, Na_V_1.8, and Na_V_1.9. Data are presented as mean ± SEM, with n = 3–9 cells per data point. (**c**) Representative hNa_V_1.1-1.9 current traces before (black) and after addition of Pn3a (red). Currents were obtained by a 20 ms pulse of −20 mV for Na_V_1.1-1.7, a 20 ms pulse +10 mV for Na_V_1.8, and a 40 ms pulse of −40 mV for Na_V_1.9. Pn3a (10 nM) selectively inhibited peak current at hNa_V_1.7 only.

Detailed subtype selectivity characterization using whole cell electrophysiology in HEK293 cells revealed that Pn3a most potently inhibits hNa_V_1.7 (pIC_50_ 9.06 ± 0.08 M), with 40-fold selectivity over hNa_V_1.1 (pIC_50_ 7.43 ± 0.06 M), 100-fold selectivity over hNa_V_1.2 (pIC_50_ 6.91 ± 0.07 M), hNa_V_1.3 (pIC_50_ 6.68 ± 0.08 M), hNa_V_1.4 (pIC_50_ 6.84 ± 0.08 M) and hNa_V_1.6 (pIC_50_ 6.89 ± 0.05 M), and greater than 900-fold selectivity over the tetrodotoxin (TTX)-resistant subtypes hNa_V_1.5 (pIC_50_ 6.10 ± 0.06 M), hNa_V_1.8 (pIC_50_ 4.30 ± 0.09 M), and hNa_V_1.9 (pIC_50_ 5.62 ± 0.11 M), making Pn3a one of the most selective Na_V_1.7 inhibitors reported to date (Fig. 2a,b and see Table 1 for IC_50_ values). Pn3a inhibited mouse Na_V_1.7 (pIC_50_ 8.36 ± 0.11 M) and rat Na_V_1.7 (pIC_50_ 8.83 ± 0.18 M) with similar potency to human Na_V_1.7, which is important for pre-clinical *in vivo* studies performed in rodents (see Table 1 for IC_50_ values). At a concentration that nearly abolished inward current at Na_V_1.7, Pn3a (10 nM) had little effect on the current traces for other Na_V_ isoforms, with the exception of enhancing inactivation at Na_V_1.1 and slowing the inactivation time constant at Na_V_1.8 (Fig. 2c). We also assessed the effects of Pn3a at selected off-targets, including voltage-gated potassium (K_V_) channels, voltage gated calcium (Ca_V_) channels and nicotinic acetylcholine receptors (nAChRs). Pn3a had no significant effect at rK_V_2.1 up to 300 nM (see below) or on hCa_V_1.2, hCa_V_2.2, α7 nAChR or α3 nAChR up to 10 μM (data not shown).

### Pn3a is a gating modifier toxin that alters the voltage-dependence of Na_V_1.7 activation

**Figure 3:**
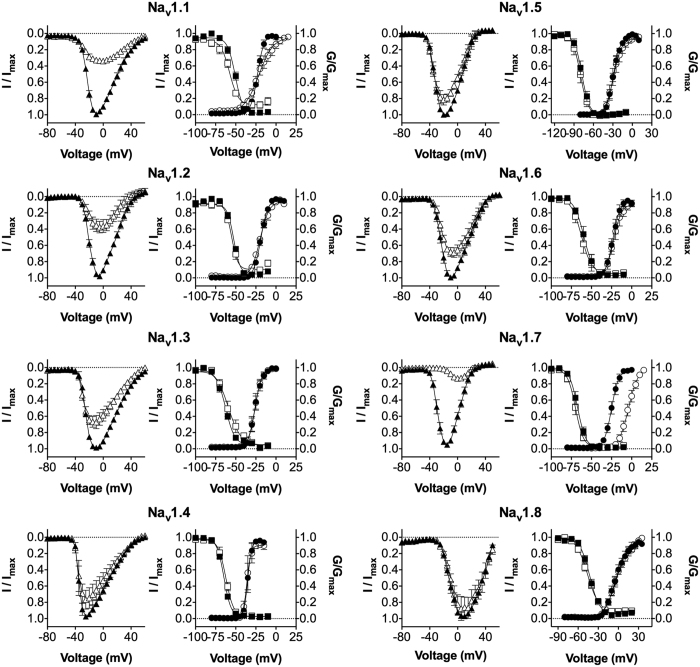
Effect of μ-TRTX-Pn3a on the electrophysiological parameters of hNa_V_1.1-1.8. I-V curves before (black triangles) and after addition of Pn3a (white triangles). Pn3a (100 nM) inhibited peak current at all Na_V_ subtypes but caused a rightward shift in the I-V curve at Na_V_1.7 only. G-V curves before (black circles) and after addition of Pn3a (white circles). Pn3a (100 nM) significantly shifted the V_1/2_ of voltage-dependence of activation to a more depolarized potential at Na_V_1.7 only (Δ + 21.3 mV). Voltage-dependence of steady-state fast inactivation curves before (black squares) and after addition of Pn3a (white squares). Pn3a (100 nM) caused small but significant shifts in the V_1/2_ of steady-state fast inactivation at Na_V_1.1, 1.2, 1.3, 1.4, 1.7 and 1.8. Data are presented as mean ± SEM, with n = 4–10 cells per data point.

**Figure 4:**
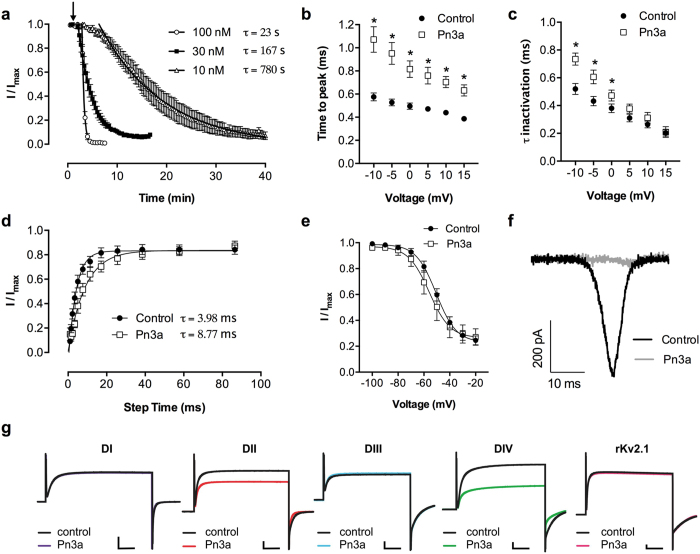
Additional effects of μ-TRTX-Pn3a on the electrophysiological parameters of hNa_V_1.7. (**a**) Time course of peak current reduction upon application of (arrow) 100 nM, 30 nM or 10 nM Pn3a at Na_V_1.7. Onset of block was measured with repetitive pulses to –20 mV every 20 s. Data are fitted with single-exponential functions and the time constant of block (τ) is indicated for each concentration. (**b**) Voltage-dependence of time-to-peak as a measure of activation kinetics at Na_V_1.7. Pn3a (100 nM) delayed time to peak between −20 mV and 15 mV. (**c**) Voltage-dependence of fast inactivation time constants at Na_V_1.7. Pn3a (100 nM) slowed the inactivation rate between −10 mV and 0 mV. (**d**) Rate of recovery from inactivation at Na_V_1.7. Pn3a (100 nM) slowed the rate of recovery from inactivation. Data are fitted with single-exponential functions and the time constant of recovery (τ) is indicated. (**e**) Steady-state slow inactivation at Na_V_1.7. Pn3a (100 nM) had no significant effect on the voltage-dependence of steady-state slow inactivation. Data are presented as mean ± SEM, with n = 4–6 cells per data point. Statistical significance was determined using two-way ANOVA, *P < 0.05 compared to control. (**f**) Representative ramp current elicited by a 50 ms depolarization from −100 to +20 mV at a rate of 2.4 mV/ms at Na_V_1.7. Pn3a (100 nM) inhibited ramp currents. (**g**) Potassium currents before (black) and in the presence of Pn3a (coloured) elicited by depolarisations to 70 mV in hNa_v_1.7/rK_v_2.1 chimeras. Pn3a (300 nM) inhibited potassium currents in the DII hNa_V_1.7/K_V_2.1 and DIV hNa_V_1.7/K_V_2.1 chimeras. Scale bars: 50 ms (abscissa), 2 μA (DI, DII, DIV, K_V_2.1) and 1 μA (DIII) (ordinate axis).

In general, spider venom-derived peptides bind to the voltage-sensing domains of Na_V_ channels to modify gating associated with channel activation and inactivation^14^. We thus examined the effect of Pn3a on the current-voltage (*I-V*) relationship as well as the voltage-dependence of activation and steady-state fast inactivation on Na_V_1.1–1.8 (Fig. 3). With Na_V_1.7, Pn3a had a slow onset of effect, with time constants of block of 23 s, 167 s and 780 s for concentrations of 100 nM, 30 nM and 10 nM, respectively (Fig. 4a). Interestingly, this slow onset of effect was only observed for Na_V_1.7 and is similar to that reported for the Na_V_1.7 selective spider peptide ProTx-II^23^. Long application times required to reach steady-state inhibition precluded testing Pn3a at lower concentrations and we thus examined the effects of 100 nM Pn3a on Na_V_1.1–1.8.

At Na_V_1.7, Pn3a (Fig. 3) shifted the voltage-dependence of activation to more depolarized membrane potentials (V_1/2_ activation: Δ = +21.3 mV), confirming gating modifier activity. Interestingly, this effect on the voltage-dependence of activation was unique to Na_V_1.7, with Pn3a (100 nM) causing no significant effect on the V_1/2_ of activation at other Na_V_ subtypes (Fig. 3 and Table 2). Minor effects were observed for Pn3a (100 nM) on the voltage-dependence of steady-state fast inactivation for Na_V_1.7 (V_1/2_ inactivation: Δ = −2.7 mV) as well as Na_V_1.1, 1.2, 1.3, 1.4 and 1.8 (Fig. 3 and Table 2).

To investigate the effects of Pn3a on the kinetics of activation and fast inactivation, we measured time-to-peak and the time constants of fast inactivation at Na_V_1.7. Pn3a (100 nM) significantly slowed the time-to-peak between −10 mV and 15 mV (*P* < 0.05, two-way ANOVA; Fig. 4b) and voltage-dependently slowed the inactivation time constants between −10 mV and 0 mV (*P* < 0.05, two-way ANOVA; Fig. 4c). Pn3a (100 nM) also significantly slowed recovery from fast inactivation at Na_V_1.7 (control, τ = 3.98 ms; Pn3a, τ = 8.77 ms; *P* < 0.05; paired *t*-test;), confirming reduced availability of toxin-bound Na_V_1.7 channels for re-activation suggested by the toxin effects on steady-state fast inactivation (Fig. 4d). In addition to fast inactivation, Na_V_1.7 channels can inactivate by slow inactivation, a process that occurs over seconds (as opposed to milliseconds) and results in a different conformational state. Pn3a (100 nM) had no effect on the voltage-dependence of steady-state slow inactivation, causing no significant shift in the V_1/2_ of slow inactivation (control, −50.5 ± 1.4 mV; Pn3a, −55.9 ± 2.9 mV; *P* > 0.05; paired *t*-test; Fig. 4e) and had no preference for the slow-inactivated state (data not shown). This is in contrast to the Na_V_1.7 selective small molecule inhibitor PF-04856264 and clinical lead PF-05089771, which both preferentially bind to the slow inactivated state^11^,^24^.

An important biophysical property of Na_V_1.7 is the ability to produce currents in response to small, slow depolarization ramps^25^. Pn3a (100 nM) completely inhibited Na_V_1.7 ramp currents elicited by a 50-ms depolarization from −100 to +20 mV (2.4 mV/ms) (Fig. 4f), indicating that it should inhibit Na_V_1.7-mediated generator currents at sensory nerve endings.

### Pn3a binds to DII and DIV of Na_V_1.7

To determine the Pn3a binding site on hNa_V_1.7, we assessed the effects of Pn3a on hNa_V_1.7/K_V_2.1 channel chimeras, where the S3-S4 linker from each domain of hNa_V_1.7 is inserted into the homotetrameric K_V_2.1 channel^26^. Pn3a (300 nM) had no effect on wild-type K_V_2.1, but inhibited potassium currents in the DII hNa_V_1.7/K_V_2.1 and DIV hNa_V_1.7/K_V_2.1 chimeras, indicating that Pn3a interacts with the S3-S4 linkers in both of these domains (Fig. 4g). This is consistent with the demonstration that interaction of Na_V_ channel toxins with any of the voltage-sensing domains I–III affect channel activation, whereas inactivation is only affected by toxins that bind *exclusively* to voltage-sensing domain IV^27^. Moreover, despite interactions with the DIV voltage-sensing domain, Pn3a potency was not significantly reduced in the hNa_V_1.7 M123 mutant (Y1537S/W1538R/D1586E) expressed in HEK293 cells (data not shown), indicating that Pn3a does not overlap with the binding site of the Na_V_1.7 selective small molecule inhibitor PF-04856264^11^.

### Solution structure of Pn3a

**Figure 5:**
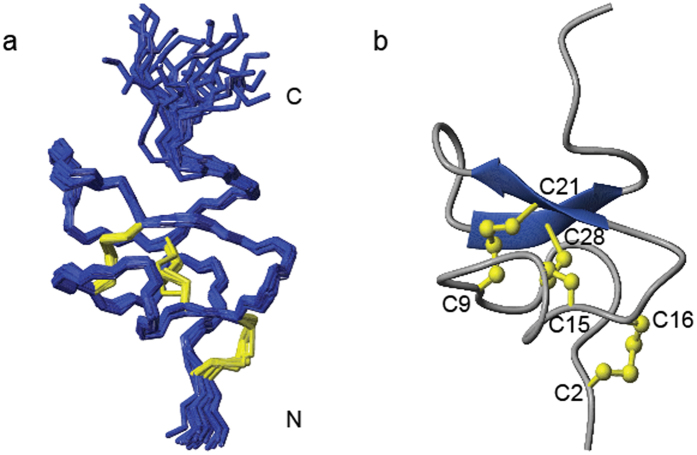
Solution structure of Pn3a. (**a**) The family of the 20 lowest energy structures superimposed over the backbone with disulfide bonds shown in yellow (Protein Data Bank code 5T4R). (**b**) The lowest energy structure with β-strands shown as arrows and disulfide bonds in ball and stick representation.

The three-dimensional solution structure of Pn3a was determined using 2D homonuclear NMR spectroscopy. The NMR data were of excellent quality with sharp and well-dispersed resonances, consistent with a single well-ordered structure in solution. NMR-derived interproton distances, hydrogen bonds and dihedral angles were used for structure calculations with simulated annealing and subsequent water minimization^28^. The ensemble of 20 conformations representing the solution structure of Pn3a is shown in Fig. 5a (Protein Data Bank code 5T4R) and the structural statistics presented in Supplementary Table 1. Pn3a forms an inhibitor cysteine knot (ICK) fold that is common in spider-venom peptides. The ICK motif provides rigidity to the peptide, with the only backbone flexibility observed at the termini (Fig. 5a). An anti-parallel β-sheet is formed between β-strands I (K19-K22) and II (R27-R27) that are connected by four tight turns (Fig. 5b). The β-sheet structure correlated with observed NOE peaks and predicted hydrogen bonding patterns and is a common structural element for peptides with an ICK^29^,^30^.

### Pn3a has on-target activity in DRG neurons and spinal slices

**Figure 6:**
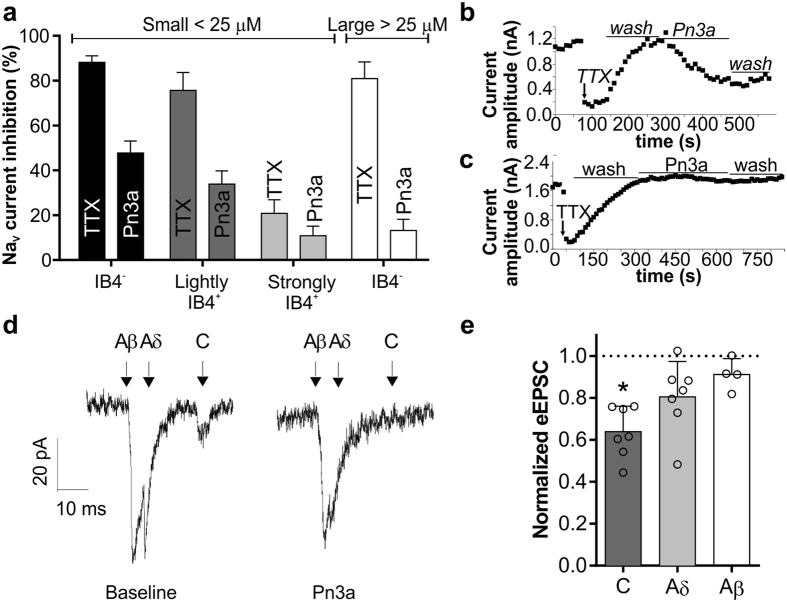
Pn3a inhibits TTX-sensitive current in small-diameter DRG neurons and eEPSC in C-fibres from lamina I neurons. (**a**) Percentage inhibition of sodium current by TTX and Pn3a in IB_4_^−^, lightly IB_4_^+^ and strongly IB_4_^+^ small-diameter and large-diameter DRG neurons (n = 6–15). (**b**) Representative peak current vs time plot before and after addition of TTX and Pn3a in a small-diameter DRG neuron. (**c**) Representative peak current vs time plot before and after addition of TTX and Pn3a in a large-diameter DRG neuron (**d**) Representative current traces from a lamina I neuron indicating the position of the dorsal root evoked Aβ-, Aδ- and C-fibre currents pre- and post-treatment with Pn3a. (**e**) eEPSC amplitudes normalized to the baseline control for C-, Aδ-, Aβ-fibre currents in lamina I neurons treated with Pn3a (n = 7, 7 and 4, respectively). Data are presented as mean ± SEM. Statistical significance was determined using *t*-test compared to control, *P < 0.05.

We assessed the effects of Pn3a on Na^+^ currents in acutely dissociated rat small-diameter dorsal root ganglion (DRG) neurons; in which Na_V_1.7 is the major TTX-sensitive channel^24^. Pn3a partially inhibited TTX-sensitive sodium current in small-diameter IB_4_^−^ neurons (% inhibition: TTX (1 μM), 88 ± 3%; Pn3a (300 nM), 48 ± 5%), small-diameter lightly IB_4_^+^ neurons (% inhibition: TTX (1 μM), 76 ± 8%; Pn3a (300 nM), 34 ± 6%) and small-diameter strongly IB_4_^+^ neurons (% inhibition: TTX (1 μM), 21 ± 6%; Pn3a (300 nM), 11 ± 4%) neurons (Fig. 6a,b). The effect of Pn3a on TTX-sensitive current in large-diameter DRG neurons was less pronounced (% inhibition: TTX (1 μM), 81 ± 7%; Pn3a (300 nM), 13 ± 5%) (Fig. 6a,c).

We also assessed the effect of Pn3a on electrically evoked excitatory post-synaptic currents (eEPSCs) in neurons of the superficial dorsal horn. Pn3a (1 μM) significantly decreased C-fibre eEPSC amplitudes in lamina I neurons (% inhibition: 36.1 ± 4.6%; n = 7, *P* < 0.05; paired *t*-test). No significant decrease in eEPSC amplitudes was observed Aδ-fibres (% inhibition: 19.4 ± 6.4%; n = 7, *P* > 0.05; paired *t*-test) or Aβ-fibres (% inhibition: 8.7 ± 3.8%; n = 4, *P* > 0.05; paired *t*-test) (Fig. 6d,e).

### Administration of selective Na_V_1.7 inhibitors alone does not produce analgesia

**Figure 7:**
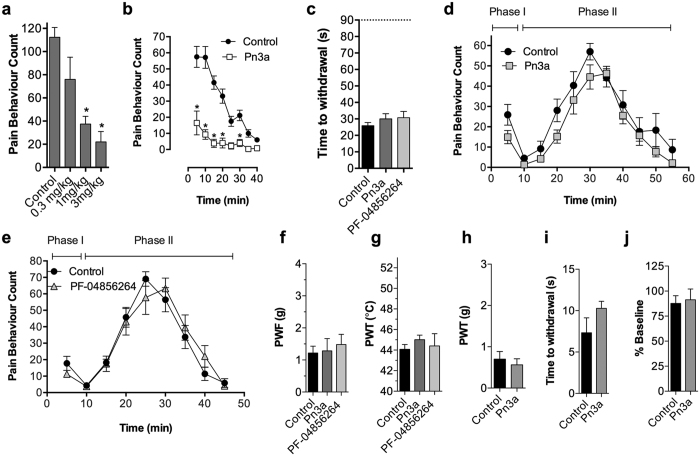
Analgesic effects of selective Na_V_1.7 inhibitors. (**a**) Pn3a (i.p.) dose-dependently reversed spontaneous pain behaviours elicited by intraplantar injection of the Na_V_1.7 activator OD1 in mice (over 10 min); n = 3–9 per group. (**b**) Time course of reversal of OD1-induced spontaneous pain behaviours by Pn3a (3 mg/kg i.p.); n = 6 per group. (**c**) Effect of Pn3a (3 mg/kg i.p.) and PF-04856264 (30 mg/kg i.p.) on noxious heat assessed using the hotplate test (50 °C) in mice; n = 6–16 per group. Dashed line represents time spent on the hotplate by Na_V_1.7^Wnt^ knockout mice. (**d**) Time course of effect of Pn3a (3 mg/kg i.p.) and (**e**) PF-04856264 (30 mg/kg i.p.) on formalin-induced spontaneous pain behaviours in mice; n = 4–15 per group. Effect of Pn3a (3 mg/kg i.p.) and PF-04856264 (30 mg/kg i.p.) on (**f**) carrageenan-induced mechanical allodynia and (**g**) carrageenan-induced thermal allodynia in mice; n = 4–12 per group. Effect of Pn3a (0.3 nmoles i.t.) on (**h**) FCA-induced mechanical allodynia, (**i**) FCA-induced thermal allodynia, and (**j**) motor performance on the Rotarod in rats; n = 4–9 per group. Data are presented as mean ± SEM. Statistical significance was determined using *t*-test, one-way or two-way ANOVA with Dunnett’s post-test as appropriate, *P < 0.05 compared to control.

To assess the analgesic potential of selective Na_V_1.7 inhibition *in vivo*, we examined the effects of Pn3a, as well as the Na_V_1.7-selective small molecule inhibitor PF-04856264, in multiple rodent models of pain. To assess on-target activity at Na_V_1.7 *in vivo*, we assessed analgesic efficacy of Pn3a in a mouse model of Na_V_1.7-mediated pain based on intraplantar injection of the scorpion toxin OD1, which impairs inactivation and enhances persistent current produced by Na_V_1.7^19^. Pn3a administered by intraperitoneal injection dose-dependently reduced OD1-induced spontaneous pain behaviours in the absence of adverse effects (pain behaviours/10 min: control, 112 ± 8; Pn3a (0.3 mg/kg), 76 ± 19; Pn3a (1 mg/kg), 37 ± 7; Pn3a (3 mg/kg), 22 ± 9; *P* < 0.05, one-way ANOVA; Fig. 7a), confirming on-target Na_V_1.7 activity *in vivo*. At the highest dose tested (3 mg/kg), analgesic activity persisted for at least 40 min, confirming Pn3a had a duration of action suitable for assessment in other pain models (Fig. 7b). We did not test PF-04856264, as efficacy in the OD1 model at a dose of 30 mg/kg was previously reported^19^.

We next tested Pn3a and PF-04856264 in mouse models of pain where genetic deletion of Na_V_1.7 attenuates or abolishes pain behaviours. Surprisingly, at doses that attenuated OD1-induced spontaneous pain, neither Pn3a or PF-04856264 reduced responses to noxious heat assessed by the hot plate test (time to withdrawal: control, 25.9 ± 1.8 s; Pn3a (3 mg/kg i.p.), 30.0 ± 3.1 s; PF-04856264 (30 mg/kg i.p.), 30.7 ± 3.8 s; *P* > 0.05, one-way ANOVA; Fig. 7c), despite noxious heat responses being abolished the Na_V_1.7^Wnt^ knockout mouse^31^. In the formalin model, where genetic deletion of Na_V_1.7 attenuates formalin-induced spontaneous behaviours in both Phase I and Phase II^31^,^32^, neither Pn3a (3 mg/kg i.p.) (Fig. 7d) or PF-04856264 (30 mg/kg ip.) (Figure 7e) significantly reduced Phase I or Phase II pain behaviours (*P* > 0.05, two-way ANOVA). Similar results were found with carrageenan-induced inflammation, where thermal allodynia is abolished in the Na_V_1.7^Nav1.8^ knockouts^33^, but Pn3a and PF-04856264 had no significant anti-allodynic effect on mechanical thresholds (paw withdrawal force; PWF: control, 1.2 ± 0.2 g; Pn3a (3 mg/kg i.p.), 1.3 ± 0.4 g; PF-04856264 (30 mg/kg i.p.), 1.5 ± 0.3 g; *P* > 0.05, one-way ANOVA; Fig. 7f) or thermal thresholds (paw withdrawal temperature; PWT: control, 44.1 ± 0.5 °C; Pn3a (3 mg/kg i.p.), 45.0 ± 0.4 °C; PF-04856264 (30 mg/kg i.p.), 44.4 ± 1.2 °C; *P* > 0.05, one-way ANOVA; Fig. 7g).

As Pn3a and PF-04856264 are unlikely to cross the blood-brain barrier^34^, we also assessed if intrathecal administration of Na_V_1.7 inhibitors is required to elicit analgesia in Freund’s Complete Adjuvant (FCA)-induced inflammation, where mechanical and thermal allodynia are abolished in Na_V_1.7^Nav1.8^ knockouts^33^. Intrathecal Pn3a (0.3 nmoles) failed to reverse FCA-induced mechanical allodynia (PWF: control, 0.7 ± 0.2 g; Pn3a, 0.6 ± 0.1 g; *P* > 0.05; unpaired *t*-test; Fig. 7h) or thermal allodynia (time to withdrawal: control, 7.3 ± 1.8 s; Pn3a, 10.3 ± 0.8 s; *P* > 0.05; unpaired *t*-test; Fig. 7i) in rats. Notably, intrathecal Pn3a (0.3 nmoles) caused no motor adverse effects consistent with high Na_V_1.7 selectivity at this dose *in vivo* (Rotarod% Baseline: control, 88 ± 7%; Pn3a, 91 ± 11%; *P* > 0.05; unpaired *t*-test; Fig. 7j); however motor impairment was observed at a higher dose of 1 nmole (data not shown). Intrathecal PF-04856264 was not tested due to low potency at the rat Na_V_1.7 channel^11^.

### Selective Na_V_1.7 inhibitors synergize with opioids to produce analgesia

Altered expression of endogenous opioid peptides was recently shown to contribute to the pain-free phenotype observed in humans and mice lacking Na_V_1.7^21^. We therefore assessed the analgesic effects of the selective Na_V_1.7 inhibitors Pn3a and PF-04856264 in combination with subtherapeutic doses of the opioid oxycodone.

**Figure 8:**
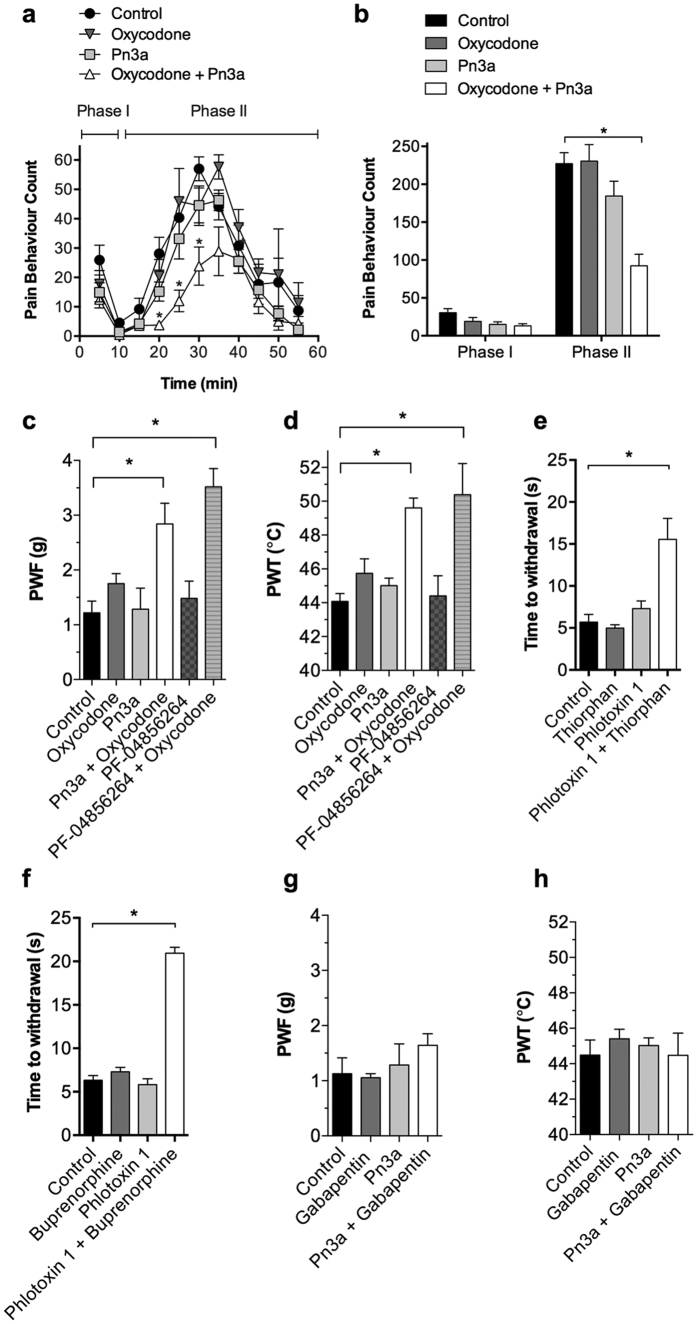
Selective Na_V_1.7 inhibitors synergize with opioids to produce analgesia. (**a**) Time course of effect and (**b**) total pain behaviours with Pn3a (3 mg/kg i.p.), oxycodone (1 mg/kg i.p.) or the combination, in Phase I (0–10 min) and Phase II (10–55 min) of the formalin model in mice; n = 7–11 per group. Effect of Pn3a (3 mg/kg i.p.), PF-04856264 (30 mg/kg i.p.), oxycodone (0.66 mg/kg i.p.), or the combinations on (**c**) carrageenan-induced mechanical allodynia and (**d**) carrageenan-induced thermal allodynia in mice; n = 4–12 per group. (**e**) Effect of Phlotoxin-1 (50 μg/kg i.p.) and thiorphan (20 mg/kg i.p.) alone or in combination on acute heat responses; n = 6 per group (**f**) Effect of phlotoxin 1 (50 μg/kg i.p.) and buprenorphine (50 μg/kg i.p.) alone or in combination on acute heat responses; n = 6 per group. Effect of Pn3a (3 mg/kg i.p.) and gabapentin (100 mg/kg i.p.) alone or in combination on (**g**) carrageenan-induced mechanical allodynia and (**h**) carrageenan-induced thermal allodynia in mice; n = 3–8 per group. Data are presented as mean ± SEM. Statistical significance was determined using one-way or two-way ANOVA with Dunnett’s post-test as appropriate, *P < 0.05 compared to control.

In the formalin model, administration of Pn3a with a subtherapeutic dose of oxycodone significantly reduced pain behaviours in Phase II only (pain behaviours: control, 227 ± 14; oxycodone (1 mg/kg i.p.), 231 ± 22; Pn3a (3 mg/kg i.p.), 185 ± 20; Pn3a (3 mg/kg i.p.) + oxycodone (1 mg/kg i.p.), 92 ± 15; *P* < 0.05, two-way ANOVA; Fig. 8a,b). Similar effects were seen in the carrageenan model, where administration of either Pn3a or PF-04856264 with a sub-therapeutic dose of oxycodone significantly increased mechanical thresholds (PWF: control, 1.2 ± 0.2 g; Pn3a (3 mg/kg i.p.), 1.3 ± 0.4 g; oxycodone (0.67 mg/kg i.p.), 1.8 ± 0.2 g; Pn3a (3 mg/kg i.p.) + oxycodone (0.67 mg/kg i.p.), 2.8 ± 0.4 g; PF-04856264 (30 mg/kg i.p.), 1.5 ± 0.3 g; PF-04856264 (30 mg/kg i.p.) + oxycodone (0.67 mg/kg i.p.), 3.5 ± 0.3 g; *P* < 0.05, one-way ANOVA; Fig. 8c) and thermal thresholds (PWT: control, 44.1 ± 0.5 °C; Pn3a (3 mg/kg i.p.), 45.0 ± 0.4 °C; oxycodone (0.67 mg/kg i.p.), 45.7 ± 0.8 °C; Pn3a (3 mg/kg i.p.) + oxycodone (0.67 mg/kg i.p.), 49.6 ± 0.6 °C; PF-04856264 (30 mg/kg i.p.), 44.4 ± 1.2 °C; PF-04856264 (30 mg/kg i.p.) + oxycodone (0.67 mg/kg i.p.), 50.4 ± 1.8 °C; *P* < 0.05, one-way ANOVA; Fig. 8d). Intraplantar administration of Pn3a (3 μM) with a sub-therapeutic dose of oxycodone did not result in analgesic synergy (PWF: 1.1 ± 0.1 g; PWT: 44.3 ± 0.6 °C). At these doses Pn3a alone or in combination with oxycodone had no significant effect on the parallel rod floor test (ataxia index: control, 3.7 ± 0.5; Pn3a, 4.3 ± 0.4; Pn3a + oxycodone, 4.2 ± 0.4; *P* > 0.05, one-way ANOVA), confirming the analgesic effects were not due to motor impairment.

To confirm the lack of efficacy wasn’t specific to Pn3a, we assessed another previously characterized spider peptide Nav1.7 inhibitor phlotoxin 1^20^, and extended our assessment of opiate synergy to buprenorphine and the enkephalinase inhibitor thiorphan. Phlotoxin 1 (sequence ACLGQWDSCDPKASKCCPNYACEWKYPW CRYKLF) inhibits Na_V_1.7 with an IC_50_ of 260 nM but has no effect on other Na_V_ isoforms at concentrations up to 2 μM^20^. Treating mice with phlotoxin 1 alone had little effect on acute heat pain-sensing responses measured with a Hargreaves apparatus. However, co-administration of phlotoxin 1 with thiorphan resulted in substantial analgesia (time to withdrawal: control, 5.7 ± 0.9 s; thiorphan (20 mg/kg i.p.), 5.0 ± 0.4 s; phlotoxin 1 (50 μg/kg i.p.), 7.3 ± 0.9 s; phlotoxin 1 (50 μg/kg i.p.) + thiorphan (20 mg/kg i.p.), 15.6 ± 2.5 s; *P* < 0.05, one-way ANOVA; Fig. 8e). Similar results were obtained when phlotoxin 1 was given in combination with the opioid agonist buprenorphine (time to withdrawal: control, 6.3 ± 0.5 s; buprenorphine (50 μg/kg i.p.), 7.2 ± 0.5 s; phlotoxin 1 (50 μg/kg i.p.), 5.8 ± 0.7 s; phlotoxin 1 (50 μg/kg i.p.) + buprenorphine (50 μg/kg i.p.), 21.0 ± 0.7 s; *P* < 0.05, one-way ANOVA; Fig. 8f), consistent with the data obtained with Pn3a. Next we assessed if the analgesic synergy was specific to opioids, or whether another clinically used analgesic, such as gabapentin, could also produce analgesia when given in combination with Pn3a. Administration of Pn3a with a subtherapeutic dose of gabapentin had no significant effect on carrageenan-induced mechanical allodynia (PWF: control, 1.1 ± 0.3 g; gabapentin (100 mg/kg i.p.), 1.1 ± 0.1 g; Pn3a (3 mg/kg i.p.) + gabapentin (100 mg/kg i.p.), 1.6 ± 0.2 g; *P* > 0.05, one-way ANOVA; Fig. 8g) or thermal allodynia (PWT: control, 44.5 ± 0.9 °C; gabapentin (100 mg/kg i.p.), 45.4 ± 0.5 °C; Pn3a (3 mg/kg i.p.) + gabapentin (100 mg/kg i.p.), 44.5 ± 1.2 °C; *P* > 0.05, one-way ANOVA; Fig. 8h).

## Discussion

Since loss-of-function mutations in *SCN9A* were first identified as the cause of CIP in 2006^4^, there has been wide interest in the development of selective Na_V_1.7 inhibitors for treatment of pain^35^. However, due to high sequence homology between Na_V_1.1–1.9, development of highly selective Na_V_1.7 inhibitors has been challenging, making assessment of analgesic potential of truly selective Na_V_1.7 inhibition difficult to ascertain. In the search for novel Na_V_ inhibitors, we screened spider venoms, a known rich source of disulfide-containing peptides with potential for subtype-selective activity at Na_V_ channels, and subsequently identified Pn3a as a novel Na_V_1.7 subtype selective inhibitor.

Characterisation of Pn3a revealed it as one of the most potent and selective Na_V_1.7 inhibitors reported to date, with 40-fold selectivity over Na_V_1.1 and at least 100-fold selectivity over all other Na_V_ subtypes. In comparison, ProTx-II, a spider peptide from the venom of *Thrixopelma pruriens* is at least 85-fold selective over Na_V_1.2–Na_V_1.8^23^ while the aryl sulfonamide PF-04856264 binds to the slow inactivated state of Na_V_1.7 with at least 65-fold selectivity and the clinical lead PF-05089771 is only at least 10-fold selective^19^,^24^. While the required level of subtype-selectivity is difficult to estimate, assuming equivalent tissue penetrance and on-target activity and Hill slope of inhibition close to unity, 100-fold selectivity should enable complete Na_V_1.7 inhibition in the absence of marked effects on the function of other isoforms. High subtype-selectivity is important, since activity at other Na_V_ isoforms, such as Na_V_1.6, likely contributes to analgesic activity but also adverse effects^36^,^37^,^38^.

Na_V_1.7 is expressed on sensory nerve endings in the skin, where it is thought to have a major role in regulating the excitability of peripheral sensory neurons^39^. The biophysical properties contributing to this specialized function include the slow closed-state inactivation that prevents Na_V_1.7 from inactivating during subthreshold depolarisations originating from transducer channels on sensory nerve endings in response to noxious stimuli. This enables Na_V_1.7 to amplify subthreshold depolarizations, or generator potentials, by producing ramp currents, which in turn cause sufficient membrane depolarization to activate Na_V_1.8, the major contributor to action potential electrogenesis in nociceptors^40^,^41^. Indeed, enhanced activation of Na_V_1.7 at peripheral nerve terminals, whether through toxins like OD1, or due to altered biophysical properties arising from gain-of-function mutations, undoubtedly leads to enhanced afferent firing and pain^5^,^6^,^7^,^42^. Thus, Na_V_1.7 at sensory nerve endings could be a key site of action for Na_V_1.7 inhibitors. Indeed, at the highest dose tested, Pn3a was able to significantly reduce spontaneous pain behaviours in mice caused by intraplantar injection of the Na_V_1.7 activator OD1, confirming on-target activity at Na_V_1.7 on sensory nerve endings *in vivo*. Although pain behaviours were not completely abolished, they were reduced to a similar level to those seen in Na_V_1.7 knockout mice, with residual pain responses likely attributable to Na_V_1.6^19^.

In addition, Pn3a showed on-target activity on small-diameter DRG neurons, evidenced by inhibition of ~50% of TTX-sensitive current in both IB_4_^+^ and IB_4_^−^ neurons, while TTX−sensitive currents remained largely unaffected in large DRG neurons. This is consistent with global Na_V_1.7 knockout mice, in which 37% of TTX-sensitive current is reduced in DRG neurons^32^. Interestingly, the majority of sodium current in small-diameter IB_4_^+^ neurons was TTX resistant, likely attributable to Na_V_1.9^43^. However, despite clear inhibition of Na_V_1.7 in sensory neurons both *in vitro* and *in vivo*, neither Pn3a nor the aryl sulfonamide PF-04856264 produced analgesia in rodent models of acute nociception or inflammatory pain at doses sufficient to reduce OD1-induced spontaneous pain. While it is clear that enhanced activation of Na_V_1.7 at peripheral nerve terminals is sufficient to drive pain^19^, this lack of efficacy in animal models suggests that acute pharmacological inhibition of Na_V_1.7 at peripheral sensory nerve endings alone is not sufficient to elicit analgesia in these models. Five Na_V_ subtypes are expressed on adult peripheral sensory neurons (Na_V_1.1, 1.6, 1.7, 1.8, 1.9), with C-fibres predominantly expressing Na_V_1.7 and the TTX-resistant subtypes Na_V_1.8 and Na_V_1.9^44^,^45^,^46^. While it is thought that Na_V_1.7 plays a major role in setting the threshold of activation, Na_V_1.9 may also be involved in the production of ramp currents and amplification of generator potentials^41^. Indeed, in global Na_V_1.7 knockout mice, the receptive fields of C-fibres are still mechanically excitable^32^, suggesting that Na_V_1.7 channels on sensory nerve endings are not solely responsible for regulating action potential generation in all nociceptive neurons.

The spider peptide ProTx-II is also reported to lack analgesic activity in a rodent model of inflammatory pain, despite reaching plasma levels sufficient to inhibit Na_V_1.7 *in vitro*^23^. ProTx-II’s lack of analgesic activity was attributed to an inability to cross the blood-nerve-barrier, a sheath of connective tissue layers that encompasses the axons of peripheral nerves, and thus an inability to inhibit action potential propagation^47^. However, while Na_V_1.7 is expressed on axons, it is unlikely to have a major role in action potential propagation, given it has slow recovery from fast inactivation (repriming) compared to other Na_V_ subtypes, such as Na_V_1.6 and Na_V_1.8, which can sustain repetitive firing^39^,^48^,^49^.

Na_V_1.7 is also expressed on the central projections of DRG neurons in the superficial laminae I and II of the spinal cord, localised on the pre-synaptic terminals, where it is thought to be important in the regulation of neurotransmitter release^24^,^31^,^39^. As the physicochemical properties of spider peptides generally preclude crossing of the blood-brain barrier, we assessed if Na_V_1.7 inhibition at the level of the spinal cord is required to produce analgesia by administering Pn3a intrathecally. Despite reducing C-fibre evoked eEPSC amplitudes in lamina I neurons by 36%, intrathecal Pn3a at the highest tolerated dose had no analgesic activity *in vivo*. This therefore suggests that a greater level of eEPSC amplitude inhibition is required to translate to analgesia *in vivo*. While it is unclear what level of Na_V_1.7 inhibition Pn3a achieved when delivered by the intrathecal route, it is clear it was active *in vivo* by the occurrence of motor adverse effects at the highest dose tested.

Pn3a inhibits Na_V_1.7 by decreasing peak current and shifting the voltage-dependence of activation to more depolarized potentials; however, current is not completely inhibited at all membrane potentials. While most mutations associated with CIP lead to a complete loss of Na_V_1.7 function, it is unclear what level of pharmacological inhibition is required to recapitulate a pain-free phenotype. Dynamic clamp experiments in small DRG neurons suggest that total loss of Nav1.7 should increase current threshold for single action potentials by about 40%, together with a marked decrease in ability to produce repetitive impulse activity^50^. Two mutations (W1775R and L1831X) have been identified in CIP patients that retain Na_V_1.7 function, but cause a voltage-dependent depolarizing shift in channel activation, similar to Pn3a^51^. This suggests that a high level of, but not complete, inhibition is sufficient to cause analgesia. However, it should be noted that these mutations would be associated with substantial loss of Na_V_1.7 current at central and peripheral sites. It is plausible that this is not achievable with peptidic inhibitors or small molecule inhibitors such as the aryl sulfonamides which penetrate the blood-brain barrier poorly. Thus, our data support evidence from genetic studies suggesting that substantial reduction of Na_V_1.7 current is required for analgesia.

Although Pn3a was able to almost completely reverse OD1-induced pain behaviours, this may represent a specialised case, with the apparent analgesic effect arising from concentration-dependent normalisation of Na_V_1.7 function. Consistent with this notion, intraperitoneal injection of Pn3a produced reversal of OD1-induced pain behaviours in a dose-dependent manner. In contrast, antinociception in naïve animals, or the inflammatory models assessed here, may require complete or near complete inhibition of Na_V_1.7 at all peripheral and central sites. It thus remains to be determined whether incomplete Na_V_1.7 inhibition will lead to partial analgesia in conditions such as IEM, where exaggerated pain develops from enhanced Na_V_1.7 function.

Thus, the most likely explanation for the lack of efficacy of Pn3a remains the lack of sufficient inhibition of Na_V_1.7, rather than a requirement for a concomitant opioid activation.

In this study, the selective Na_V_1.7 inhibitors Pn3a, PF-04856264 and phlotoxin 1 were administered once as a single dose. It is unclear if repeated administration or chronic administration of Na_V_1.7 inhibitors is required to elicit analgesia. While limited material and poor pharmacokinetics precluded testing Pn3a chronically, it is interesting to note that the pain-free phenotype develops over several days in inducible Na_V_1.7 knockout mice^52^. While this could reflect slow turn-over of Na_V_1.7, an alternative explanation could be that analgesia emerges in parallel with upregulation of the endogenous opioid precursor proenkephalin (Penk), an effect unlikely to be reproduced with single administration of a Na_V_1.7 inhibitor with a relatively short duration of action^21^.

In further support of an important link between Na_V_1.7 and opioids, we were able to produce analgesia by administering the selective Na_V_1.7 inhibitors Pn3a, PF-04856264 and phlotoxin 1 in combination with a sub-effective dose of the clinically used opioids oxycodone or buprenorphine or the enkephalinase inhibitor thiorphan, but not with the calcium channel inhibitor gabapentin. While these data clearly demonstrate that analgesic synergy between Na_V_1.7 inhibitors and opioids is not restricted to a single agent or single pharmacological class, the mechanisms underlying synergistic analgesia remain unclear. Interestingly, the opioid antagonist naloxone substantially reverses analgesia in both mice and human lacking functional Na_V_1.7^21^. In the transcriptome of sensory neurons from Advillin-Cre Na_V_1.7 knockout mice, no changes in endogenous opioid receptor transcripts were reported, only upregulation of Penk mRNA and Met-Enkephalin protein^21^. It therefore appears that Na_V_1.7 inhibition in combination with opioid receptor activation, whether through upregulation of endogenous opioids, such as the enkephalins as demonstrated in the knockout experiments, or administration of exogenous opioids, as described here, can elicit profound analgesia^53^. Opioid receptors are expressed on peripheral sensory neurons, both on the peripheral and central projections, as well as along the axon, where they are involved in regulating neuronal excitability, action potential propagation, and neurotransmitter release through inhibition of calcium currents^54^,^55^. Thus, the synergistic activity of opioid receptor agonists with selective Na_V_1.7 inhibitors may involve both central and/or peripheral opioid effects. Interestingly, analgesic synergy between oxycodone and Pn3a was not observed after intraplantar administration, suggesting that inhibition of Na_V_1.7 at the receptive fields does not contribute to the synergistic effect observed after systemic dosing.

In summary, based on its exquisite selectivity, Pn3a is a useful pharmacological tool to probe the role of Na_V_1.7 inhibition in pain. While efficacy in the OD1 model, as well as emerging positive clinical trial results with small molecule aryl sulfonamides^56^, suggest that Na_V_1.7 inhibitors may be efficacious in IEM or PEPD, our results reveal that acute administration of peripherally restricted Na_V_1.7 inhibitors alone may not have broad efficacy in inflammatory pain. The efficacy of peripherally restricted Na_V_1.7 inhibitors in other pain types, such as neuropathic pain, remains to be determined, and underscores the need for further studies to determine how, or whether, Nav1.7 blockade will reduce pain in humans. Our results do highlight that the combination of Na_V_1.7 inhibitors and opioids may provide a novel therapeutic approach for the treatment of pain.

## Methods

### Isolation of Pn3a

Crude venom was isolated from a single specimen of *Pamphobeteus nigricolor* by electrostimulation as previously described^57^. Crude venom (1 mg) was dissolved in 5% acetonitrile (ACN)/0.1% formic acid and loaded onto an analytical C_18_ reversed-phase (RP) HPLC column (Vydac 4.6 × 250 mm, 5 μm; Grace, Columbia, MD, USA) attached to an UltiMate 3000 HPLC system (Dionex, Sunnyvale, CA, USA). Components were eluted at 0.7 mL/min and collected into 1 min fractions with solvent A (99.9% H_2_O, 0.1% formic acid) and solvent B (90% ACN, 0.1% formic acid in H_2_O) using the following gradient: 5% solvent B over 5 min, followed by 5–50% solvent B over 45 min followed by 50–100% solvent B over 15 min.

Venom fractions were assessed for activity at rNa_V_1.3 stably expressed in HEK293 cells using a FLIPR^TETRA^ (Molecular Devices) membrane potential assay as previously described^58^. Active fractions were further fractionated to near-purity and peptide masses were determined using matrix-assisted laser desorption/ionization time-of-flight (MALDI-TOF) MS using a Model 4700 Proteomics Analyser (Applied Biosystems, Foster City, CA, USA) by spotting HPLC fractions with α-cyano-4-hydroxycinnamic acid (7 mg/mL in 50% ACN). Peptides were then reduced and alkylated and N-terminal sequence determination was performed by the Australian Proteome Analysis Facility (Macquarie University, NSW, Australia).

### Synthesis of Pn3a

μ-TRTX-Pn3a was assembled on a Symphony authomated peptide synthesiser (Protein Technologies Inc., Tucson, AZ, USA) peptide synthesiser using a H-Thr(tBu)-2-ClTrt polystyrene resin (loading 0.67 mmol/g) on a 0.1 mmol scale. Fmoc deprotection was achieved using 30% piperidine/N, N-dimethylformamide (DMF) (1 × 1.5 min, then 1 × 4 min). Couplings were performed in DMF using 5 equivalents of Fmoc-amino acid/HBTU/DIEA (1:1:1) relative to resin loading for 2 × 20 min. Amino acid side-chains were protected as Asp(OtBu), Arg(Pbf), Cys(Trt), Glu(OtBu), His(Trt), Lys(Boc), Thr(tBu), Trp(Boc), Tyr(tBu). Cleavage from the resin and removal of side-chain protecting groups was achieved by treatment with 95% TFA/2.5% triisopropylsilane (TIPS)/2.5% H_2_O at room temperature for 2 h. After most of the cleavage solution was evaporated under a stream of N_2_, the product was precipitated and washed with cold diethyl ether and lyophilised from 50% ACN/0.1% TFA/H_2_O. Oxidative folding was carried out in the presence of oxidized and reduced glutathione at 4 °C and the single major product was isolated by preparative RP-HPLC.

### Cell Culture

HEK293 cells heterologously expressing human Na_V_1.1–1.7 (SB Drug Discovery, Glasgow, UK) were cultured in MEM containing 10% v/v FBS and selection antibiotics as recommended by the manufacturer. CHO cells stably expressing human Na_V_1.8 in a tetracycline-inducible system (ChanTest, Cleveland, OH) were cultured in Ham’s F-12 containing 10% (v/v) FBS and selection antibiotics as recommended by the manufacturer. To induce hNa_V_1.8 expression, cells were cultured in the presence of tetracycline (1 μg/ml) for 24 h at 27 °C. HEK293 cells expressing rNa_V_1.3^59^ or rNa_V_1.7^48^ were cultured in DMEM containing 10% v/v FBS and the selection antibiotic G-418 (0.5 mg/mL). SH-SY5Y human neuroblastoma cells were cultured in RPMI containing 15% FBS and supplemented with L-glutamine. Cells were grown in a humidified 5% CO_2_ incubator at 37 °C, grown to 70–80% confluence, and passaged every 3–4 days using TrypLE Express (Invitrogen). HEK293 cells heterologously expressing human Na_V_1.9, mouse Na_V_1.7 or hNa_V_1.7 M123 mutant (Y1537S/W1538R/D1586E)^11^ were generated and cultured by Icagen Inc (Durham, NC, USA).

### Calcium responses in SH-SY5Y cells

To assess activity at Ca_V_1.3, Ca_V_2.2, α3-containing and homopentameric α7 nAChR endogenously expressed in SH-SY5Y human neuroblastoma cells, the effect of Pn3a on Ca^2+^ responses was assessed using a FLIPR^Tetra^ assay as described^60^. In brief, SH-SY5Y cells plated at a density of 50,000 cells/well on black-walled 384-well imaging plates were loaded with Calcium 4 no-wash dye (Molecular Devices) for 30 min after 48 h in culture. After 5 min pre-treatment with Pn3a (0.1–10 μM), responses were elicited by stimulation with KCl (90 mM)/CaCl_2_ (5 mM) in the presence of ω-conotoxin CVID (10 μM; Ca_V_1.3); KCl (90 mM)/CaCl_2_ (5 mM) in the presence of nifedipine (10 μM; Ca_V_2.2); nicotine (30 μM, α3-containing nAChR) or choline (30 μM) in the presence of PNU120596 (α7 nAChR). Fluorescence responses were normalized to baseline and the maximum increase in fluorescence determined using ScreenWorks 3.2.0.14 (Molecular Devices).

### Whole-cell patch-clamp electrophysiology

For hNa_V_1.1–1.8 and rNa_V_1.7, whole-cell patch-clamp experiments were performed on a QPatch-16 automated electrophysiology platform (Sophion Bioscience, Ballerup, Denmark) as described^61^. The extracellular solution contained in mM: NaCl 145, KCl 4, CaCl_2_ 2, MgCl_2_ 1, HEPES 10 and glucose 10; pH 7.4; osmolarity 305 mOsm. The intracellular solution contained in mM: CsF 140, EGTA/CsOH 1/5, HEPES 10 and NaCl 10; pH 7.3 with CsOH; osmolarity 320 mOsm. Pn3a was diluted in extracellular solution with 0.1% BSA at the concentrations stated. All Pn3a effects were compared to pre-toxin control parameters within the same cell, and incubation times were varied between 5 and 40 min depending on the time required for steady-state inhibition to be achieved.

Concentration-response curves were obtained with a holding potential of −80 mV followed by a pre-pulse of −120 mV for 100 ms and then a 20 ms test pulse of −20 mV for Na_V_1.1−1.7 and +10 mV for Na_V_1.8 (repetition interval 20 s). *I-V* curves were obtained with a holding potential of −80 mV followed by a pre-pulse of −100 mV for 50 ms and a series of 50 ms step pulses that ranged from −80 to +60 mV in 5-mV increments before returning to a holding potential of −80 mV (repetition interval 5 s). Conductance-voltage curves were obtained by calculating the conductance (*G*) at each voltage (*V*) using the equation *G* = *I*/(*V* − *V*_*rev*_), *w*here *V*_*rev*_ is the reversal potential and were fitted with a Boltzmann equation. Voltage dependence of steady-state fast inactivation was measured using a series of 500 ms pre-pulses, ranging from −120 to −10 mV in 10-mV increments, followed by a 20 ms pulse of −20 mV for Na_V_1.1–1.7 and +10 mV for Na_V_1.8 to assess the available non-inactivated channels (repetition interval 30 s). Time-to-peak was measured from pulse onset to maximal current and fast inactivation time constants were calculated by fitting current decay traces with a single exponential function using the *I-V* protocol described above. Recovery from fast inactivation was examined using a two-pulse protocol consisting of a depolarizing pulse to 0 mV for 200 ms to inactivate channels, followed by a step to −90 mV of variable duration (1–90 ms) to promote recovery, and a 20 ms test pulse to 0 mV to assess availability of channels. Voltage dependence of steady-state slow inactivation was measured using a series of 15 s pre-pulses, ranging from −100 to −20 mV in 10-mV increments, followed by a 50 ms step to −100 mV to remove fast inactivation, and a 50 ms test pulse to 0 mV to assess the available non-inactivated channels (repetition interval 30 s). Ramp currents were evoked by a depolarization from a holding potential of −100 to +20 mV at a rate of 2.4 mV/ms.

For hNa_V_1.9, mNa_V_1.7 and hNa_V_1.7 M123 mutant, whole-cell patch-clamp experiments were performed by Icagen Inc on a PatchXpress automated electrophysiology platform (Molecular Devices, Sunnyvale, CA, USA). The extracellular solution contained in mM: NaCl 135, KCl 5.4, CaCl_2_ 2, MgCl_2_ 1, HEPES 10 and glucose 5; pH 7.4; osmolarity 300 mOsm. The intracellular solution contained in mM: CsF 135, EGTA 5, CsCl 10, HEPES 10 and NaCl 5; pH 7.4; osmolarity 298 mOsm. For hNa_V_1.9, concentration-response curves were obtained with a holding potential of −140 mV followed by a 40 ms test pulse of −40 mV (repetition interval 20 s). For mNa_V_1.7 and hNa_V_1.7 M123 mutant, concentration-response curves were obtained with a holding potential of −120 mV followed by a 20 ms test pulse of 0 mV (repetition interval 10 s).

### hNa_v_1.7/rK_v_2.1 chimeras and electrophysiology

hNa_v_1.7/rK_v_2.1 chimeras containing the S3–S4 paddle regions of the four domains of hNa_v_1.7 were described previously^26^. hNa_v_1.7/rK_v_2.1 chimera or rK_v_2.1 cDNA was injected into *Xenopus laevis* oocytes, which were then held at 17 °C in ND96 solution (in mM: 96 NaCl, 2 KCl, 5 HEPES, 1 MgCl_2_, 1.8 CaCl_2_ and 50 μg·mL^−1^ gentamycin, pH 7.6) for 2–4 days before experiments. Currents were measured using two-electrode voltage-clamp electrophysiology (Axoclamp 900 A, Molecular Devices, Sunnyvale, CA, USA; 40 μL recording chamber). Micro-electrodes were filled with 3 M KCl and had resistances of 0.3–1 MΩ. Data were filtered at 4 kHz and digitized at 20 kHz using pClamp software (Molecular Devices). The external recording solution contained (in mM) 50 KCl, 50 NaCl, 5 HEPES, 1 MgCl_2_, 0.3 CaCl_2_, pH 7.6 with NaOH, and recordings were performed at room temperature (22 °C). Pn3a (300 nM) was diluted in external recording solution with 0.1% BSA. Currents were elicited by depolarisation to +70 mV from a holding potential of −90 mV (−120 mV for DIII), with a tail voltage at −60 mV (−90 mV for DIII).

### NMR structure determination

All NMR spectroscopy experiments were performed on a Bruker Avance 600 MHz spectrometer equipped with a cryoprobe. Samples for NMR spectroscopy were prepared by dissolving peptide in either 90% H_2_O/10% D_2_O or 100% D_2_O at pH of 4.0 to a final concentration of 2 mg/mL. Homonuclear ^1^H-^1^H TOCSY, NOESY and E.COSY, as well as heteronuclear natural abundance ^1^H-^15^N HSQC, and ^1^H-^13^C HSQC spectra were all recorded at 25 °C.

Following resonance assignments the NOESY spectra were assigned automatically using CYANA, and preliminary structures calculated using torsion angle dynamics^62^. Backbone and side chain dihedral angles restraints were determined from TALOS-N^63^. ^1^H-^1^H TOCSY spectra were recorded at temperatures of 15–35 °C to calculate amide temperature coefficients. Backbone amide protons that showed both slow solvent exchange in 100% D_2_O and had a chemical shift temperature dependence of more than −0.0046 parts per million (ppm)/K were considered to be definitively hydrogen bonded^64^, and included as restraints. Final structures were calculated using water refinement within the CNS program^28^. The final ensemble of 20 structures was chosen based on final energies and stereochemistry as assessed by MolProbity^65^. Structures were visualized using MolMol^65^,^66^.

### DRG preparation and electrophysiology

Spinal level L3–L5 DRGs were removed from male Sprague–Dawley rats (aged 3–6 weeks) and placed in ice-cold HEPES-buffered saline (HBS) composed of (in mM): 154 NaCl, 2.5 KCl, 1.8 CaCl_2_, 1.5 MgCl_2_, 10 HEPES, and 10 glucose (pH 7.4, 330 ± 5 mOsm). DRG cells were then prepared as described previously^67^. To visualize IB4 binding, isolated DRG cells were pre-treated with 1 μg/mL Alexa Fluor 488-conjugated *Bandeiraea simplicifolia* IB4 (Invitrogen) for 5 min at room temperature and washed with HBS for 5 min before fluorescence was examined on the inverted microscope (Olympus, IX50) used for patch-clamp recordings. Cell diameter was determined using a micrometer graticule: <25 μm was defined as small-diameter and >25 μm was defined as large-diameter.

Whole-cell patch clamp recordings were performed at room temperature (22–24 °C) using an EPC-9 patch-clamp amplifier and corresponding PULSE software from HEKA Electronik (Lambrecht/Pfalz, Germany) or an Axopatch ID amplifier (Axon Instruments, Foster City, CA, USA) using pCLAMP acquisition software (v. 5.5, Axon Instruments). Currents were sampled at 50 kHz. Patch pipettes were pulled from borosilicate glass (AM Systems, Everett, WA, USA) and pipette input resistance was 2–3 MΩ. The capacitance of individual cells ranged between 5 and 60 pF with a series resistance between 1 and 9 MΩ. Series resistance compensation of at least 80% was used in all experiments. Capacitance transients were compensated automatically using a built-in procedure of the HEKA amplifier. Leak current was subtracted online using a *P/*6 protocol. The liquid junction potential was 9 mV.

To isolate *I*_Na_, the extracellular solution contained (in mM): 110 tetraethylammonium chloride (TEACl), 30 NaCl, 2.5 KCl, 1.8 CaCl_2_, 1.2 MgCl_2_, 10 HEPES, 10 glucose, 0.1 CdCl_2_; pH 7.4; 330 ± 5 mOsm with 0.1% BSA and the intracellular pipette solution contained (in mM): 120 CsCl, 10 HEPES, 10 EGTA, 2 CaCl_2_, 5 MgATP, 5 Na_2_GTP, 5 NaCl; pH 7.3; 285 ± 5 mOsm. Peak *I*_Na_ was determined by stepping the membrane potential from a holding potential of −80 mV to 0 mV for 10 ms every 10 s. Cells were perfused with TTX (1 μM) and Pn3a (300 nM) via a pressure driven perfusion system (AutoMate Scientific). All Pn3a effects were compared to pre-toxin control parameters within the same cell.

### Spinal cord slice preparation and electrophysiology

Male Sprague-Dawley rats (aged 5–7 weeks) were anaesthetized with isoflurane, decapitated and the lumbar region of the spinal cord was removed. Parasagittal slices (440 μm thick) of spinal cord with dorsal roots intact were prepared as described previously^68^. Slices were transferred to a recording chamber and superfused continuously at 2 ml/min with normal artificial cerebro-spinal fluid (ACSF) composed of (in mM): 125 NaCl, 2.5 KCl, 1.25 NaH_2_PO_4_, 1.2 MgCl_2_, 2.5 CaCl_2_, 25 glucose, and 11 NaHCO_3_; that had been equilibrated with 95% O_2_ and 5% CO_2_ and recordings were performed at room temperature. Dodt-contrast optics was used to identify lamina I neurons in the superficial dorsal horn. A Cs^+^-based internal solution, which should minimise postsynaptic effects, was used to record electrically evoked eEPSCs and contained (in mM): 140 CsCl, 10 EGTA, 5 HEPES, 2 CaCl_2_, 2 MgATP, 0.3 NaGTP, 5 QX-314.Cl, and 0.1% biocytin (osmolarity 285–295 mOsm). Patch electrodes had resistances between 3 and 5 MΩ. Synaptic currents were measured in whole-cell voltage-clamp (−70 mV, not corrected for a liquid junction potential of 4 mV) from lamina I cells. Bipolar tungsten electrodes placed at the end of the dorsal root (≥5 mm from the entry zone) were used to elicit eEPSCs. Prior to the start of each experiment, protocols with trains of stimuli at 1 Hz, 2 Hz and 20 Hz were used to determine the presence and onset latency of C, Aδ and Aβ currents, respectively. In four experiments, strychnine (0.3 μM) was added to the ACSF to increase the likelihood of neurons with Aβ currents. Recordings were performed on neurons before and after perfusion with Pn3a (1 μM).

### Animals

For behavioural assessment we used adult male C57BL/6 J mice or Sprague Dawley rats aged 5–8 weeks. Animals were housed in groups of 2–4 per cage, under 12 h light-dark cycles with standard rodent chow and water *ad libitum*. All assessments were performed by a blinded investigator unaware of the treatment each animal received. Ethical approval for *in vivo* experiments in animals was obtained from the University of Queensland, University of Sydney or United Kingdom Home Office animal ethics committee. Experiments involving animals were conducted in accordance with the *International Association for the Study of Pain Guidelines for the Use of Animals in Research* and the *Australian Code of Practice for the Care and Use of Animals for Scientific Purposes*, 8th edition (2013).

### OD1

The Na_V_1.7 activator OD1 (300 nM) was administered by intraplantar (i.pl) injection in mice as previously described^19^. Pn3a (0.3, 1, 3 mg/kg) was diluted in saline and pre-administered 10 min prior by intraperitoneal (i.p.) injection. Spontaneous pain behaviours were counted by a blinded observer from video recordings.

### Acute Heat Responses

Mice, pre-administered Pn3a (i.p. 3 mg/kg) or PF-04856264 (i.p. 30 mg/kg), were placed on a temperature-controlled Peltier plate (Hot/Cold Plate, Ugo Basile, Comerio, Italy) set at 50 °C, and the time taken to observe a nociceptive response (hind paw lick, flinch or jump) was recorded. Paw-withdrawal latency in mice pre-administered phlotoxin 1 (i.p. 50 μg/kg; Venomtech, Valbonne, France), thiorphan (i.p. 20 mg/kg), buprenorphine (i.p. 50 μg/kg), or combinations, was determined using the Hargreaves apparatus (Plantar Analgesia Meter, IITC, CA, USA), with three withdrawal responses measured per animal.

### Formalin

Formalin (1% v/v) was administered by i.pl injection in mice as previously described^61^. Pn3a (i.p. 3 mg/kg), oxycodone (i.p. 1 mg/kg), alone or in combination, were diluted in saline and administered at the same time as formalin. Spontaneous pain behaviours were counted from video recordings. Phase I was defined as 0–10 min and Phase II defined as 10–55 min following formalin injection.

### Carrageenan

Carrageenan (1% w/v) was administered by i.pl injection in mice as previously described^42^. Behavioural assessment was performed 3 h post-injection. Pn3a (i.p. 3 mg/kg), PF-04856264 (i.p. 30 mg/kg), oxycodone (i.p. 0.67 mg/kg), gabapentin (i.p. 100 mg/kg) or the combination thereof, were administered 10 min prior to behavioural assessment. Mechanical thresholds were determined using electronic von Frey (MouseMet Electronic von Frey, TopCat Metrology, UK) as previously described^42^. Thermal thresholds were determined using the thermal probe test (MouseMet Thermal, TopCat Metrology) as described^42^.

### Freund’s Complete Adjuvant

FCA was administered undiluted in a volume of 150 μL by i.pl injection in rats. Behavioural assessment was performed 3 days post-injection of FCA. For intrathecal administration, a polyethylene lumbar catheter was inserted into the intrathecal space between vertebrae L4–L5. Pn3a (0.3 nmoles) was diluted in saline and administered by intrathecal injection in a volume of 10 μL 30 min prior to behavioural assessment. Mechanical paw withdrawal thresholds were determined using manual von Frey with a series of calibrated filaments 0.4–15.1 g (Stoelting Co., Wood Dale, USA) using the up-down method^69^. Paw withdrawal latency to a thermal stimulus was determined using the Hargreaves test (Ugo Basile, Camerio VA, Italy). Motor co-ordination was assessed using an accelerating rotarod (Ugo Basile). Latency to fall was recorded in seconds, with a maximal cut off of 300 s. At the completion of experiments, catheter placement was checked by injection of lignocaine (2%, 10 μL) and observation of rapid bilateral hind limb paralysis.

### Parallel Rod Floor Test

Motor performance in mice was assessed using the Parallel Rod Floor Test and analysed using ANY-Maze software (Stoelting Co) as described^19^. Pn3a (3 mg/kg), alone or in combination with oxycodone (0.67 mg/kg), was administered by the i.p. route as described above 15 min prior to assessment of motor performance. The ataxia index was calculated by dividing the number of foot slips by the distance travelled (m).

### Data analysis

Data were plotted and analyzed using GraphPad Prism, version 6.0. For concentration-response curves, a four-parameter Hill equation with variable Hill coefficient was fitted to the data. IC_50_ values are mean ± SEM of the negative logIC_50_ (pIC_50_). Statistical significance was defined as *P* < 0.05 and was determined by an paired *t*-test assuming equal variance, or one-way ANOVA with Dunnett’s post-test, or two-way ANOVA with Dunnett’s or Sidak’s post-test, as appropriate. Data are presented as mean ± SEM.

## Additional Information

**How to cite this article**: Deuis, J. R. *et al*. Pharmacological characterisation of the highly Na_V_1.7 selective spider venom peptide Pn3a. *Sci. Rep.* **7**, 40883; doi: 10.1038/srep40883 (2017).

**Publisher's note:** Springer Nature remains neutral with regard to jurisdictional claims in published maps and institutional affiliations.

## Supplementary Material

Supplementary Information

## Figures and Tables

**Table 1:** Potency of Pn3a at multiple ion channels.

Channel	IC_50_ (nM)
hNa_V_1.1	37
hNa_V_1.2	124
hNa_V_1.3	210
hNa_V_1.4	144
hNa_V_1.5	800
hNa_V_1.6	129
hNa_V_1.7	0.9
rNa_V_1.7	1.5
mNa_V_1.7	4.4
hNa_V_1.8	49888
hNa_V_1.9	2427
rK_V_2.1	>300
hCa_V_1.2	>10000
hCa_V_2.2	>10000
α7 nAChR	>10000
α3 nAChR	>10000

**Table 2:** Effects of Pn3a (100 nM) on Na_V_ channel voltage dependence of activation and steady-state fast inactivation.

	Pn3a	Activation		Inactivation	
		V_1/2_ (mV)	*k* (mV)	V_1/2_ (mV)	*k* (mV)
Na_V_1.1	−	−22.9 ± 0.3	4.0 ± 0.3	−50.7 ± 0.5	−4.8 ± 0.5
	+	−21.1 ± 1.1	8.8 ± 1.0*	−57.8 ± 1.0*	−5.4 ± 0.9
Na_V_1.2	−	−18.8 ± 0.3	4.4 ± 0.2	−52.6 ± 0.6	−4.0 ± 0.5
	+	−19.8 ± 0.4	5.6 ± 0.4*	−55.2 ± 0.9*	−3.9 ± 0.6
Na_V_1.3	−	−26.0 ± 0.5	4.2 ± 0.4	−62.6 ± 0.6	−5.6 ± 0.5
	+	−26.2 ± 0.5	4.2 ± 0.4	−59.4 ± 1.2*	−7.9 ± 1.1
Na_V_1.4	−	−35.1 ± 0.5	2.4 ± 0.5	−64.7 ± 0.2	−4.4 ± 0.1
	+	−36.1 ± 0.7	2.4 ± 0.6	−62.3 ± 0.6*	−4.8 ± 0.5
Na_V_1.5	−	−29.3 ± 0.4	5.4 ± 0.3	−77.7 ± 0.7	−5.7 ± 0.6
	+	−29.1 ± 0.9	6.5 ± 0.8	−80.1 ± 0.9	−6.0 ± 0.8
Na_V_1.6	−	−23.4 ± 0.6	4.5 ± 0.5	−59.4 ± 1.0	−6.2 ± 0.9
	+	−22.6 ± 0.9	5.0 ± 0.7	−62.5 ± 1.5	−6.4 ± 1.3
Na_V_1.7	−	−25.1 ± 0.5	4.5 ± 0.4	−67.2 ± 0.7	−5.2 ± 0.6
	+	−3.8 ± 0.8*	6.0 ± 0.6	−69.9 ± 0.7*	−4.9 ± 0.7
Na_V_1.8	−	−4.4 ± 0.8	8.1 ± 0.7	−42.6 ± 0.7	−7.2 ± 0.6
	+	−3.0 ± 0.7	9.1 ± 0.7	−45.9 ± 0.8*	−8.4 ± 0.7

Data are reported as mean ± SEM. *Indicates *P* < 0.05 compared to control using paired *t*-test.

## References

[b1] Catterall W. A., Goldin A. L. & Waxman S. G. International Union of Pharmacology. XLVII. Nomenclature and structure-function relationships of voltage-gated sodium channels. Pharmacol. Rev. 57, 397–409, doi: 10.1124/pr.57.4.4 (2005). 16382098

[b2] Yu F. H. & Catterall W. A. Overview of the voltage-gated sodium channel family. Genome Biol. 4, 207 (2003). 12620097 10.1186/gb-2003-4-3-207 PMC153452

[b3] Goldberg Y. P. . Loss-of-function mutations in the Nav1.7 gene underlie congenital indifference to pain in multiple human populations. Clinical genetics 71, 311–319, doi: 10.1111/j.1399-0004.2007.00790.x (2007). 17470132

[b4] Cox J. J. . An SCN9A channelopathy causes congenital inability to experience pain. Nature 444, 894–898, doi: 10.1038/nature05413 (2006). 17167479 PMC7212082

[b5] Fertleman C. R. . SCN9A mutations in paroxysmal extreme pain disorder: allelic variants underlie distinct channel defects and phenotypes. Neuron 52, 767–774, doi: 10.1016/j.neuron.2006.10.006 (2006). 17145499

[b6] Yang Y. . Mutations in SCN9A, encoding a sodium channel alpha subunit, in patients with primary erythermalgia. J. Med. Genet. 41, 171–174 (2004). 14985375 10.1136/jmg.2003.012153 PMC1735695

[b7] Cummins T. R., Dib-Hajj S. D. & Waxman S. G. Electrophysiological properties of mutant Nav1.7 sodium channels in a painful inherited neuropathy. J. Neurosci. 24, 8232–8236, doi: 10.1523/jneurosci.2695-04.2004 (2004). 15385606 PMC6729696

[b8] Trimmer J. S., Cooperman S. S., Agnew W. S. & Mandel G. Regulation of muscle sodium channel transcripts during development and in response to denervation. Dev. Biol. 142, 360–367 (1990). 2175278 10.1016/0012-1606(90)90356-n

[b9] Rogart R. B., Cribbs L. L., Muglia L. K., Kephart D. D. & Kaiser M. W. Molecular cloning of a putative tetrodotoxin-resistant rat heart Na+ channel isoform. Proc. Natl. Acad. Sci. USA 86, 8170–8174 (1989). 2554302 10.1073/pnas.86.20.8170 PMC298237

[b10] Caldwell J. H., Schaller K. L., Lasher R. S., Peles E. & Levinson S. R. Sodium channel Na(v)1.6 is localized at nodes of ranvier, dendrites, and synapses. Proc. Natl. Acad. Sci. USA 97, 5616–5620, doi: 10.1073/pnas.090034797 (2000). 10779552 PMC25877

[b11] McCormack K. . Voltage sensor interaction site for selective small molecule inhibitors of voltage-gated sodium channels. Proceedings of the National Academy of Sciences of the United States of America 110, E2724–2732, doi: 10.1073/pnas.1220844110 (2013). 23818614 PMC3718154

[b12] Ahuja S. . Structural basis of Nav1.7 inhibition by an isoform-selective small-molecule antagonist. Science 350, aac5464, doi: 10.1126/science.aac5464 (2015). 26680203

[b13] King G. F. & Hardy M. C. Spider-venom peptides: structure, pharmacology, and potential for control of insect pests. Annu. Rev. Entomol. 58, 475–496, doi: 10.1146/annurev-ento-120811-153650 (2013). 23020618

[b14] Klint J. K. . Spider-venom peptides that target voltage-gated sodium channels: pharmacological tools and potential therapeutic leads. Toxicon 60, 478–491, doi: 10.1016/j.toxicon.2012.04.337 (2012). 22543187

[b15] Zlotkin E. The insect voltage-gated sodium channel as target of insecticides. Annu. Rev. Entomol. 44, 429–455, doi: 10.1146/annurev.ento.44.1.429 (1999). 9990721

[b16] Loughney K., Kreber R. & Ganetzky B. Molecular analysis of the para locus, a sodium channel gene in Drosophila. Cell 58, 1143–1154 (1989). 2550145 10.1016/0092-8674(89)90512-6

[b17] King G. F., Escoubas P. & Nicholson G. M. Peptide toxins that selectively target insect Na(V) and Ca(V) channels. Channels (Austin, Tex.) 2, 100–116 (2008). 10.4161/chan.2.2.6022 18849658

[b18] Bosmans F. & Swartz K. J. Targeting voltage sensors in sodium channels with spider toxins. Trends Pharmacol. Sci. 31, 175–182, doi: 10.1016/j.tips.2009.12.007 (2010). 20097434 PMC2847040

[b19] Deuis J. R. . Analgesic Effects of GpTx-1, PF-04856264 and CNV1014802 in a Mouse Model of NaV1.7-Mediated Pain. Toxins (Basel) 8, doi: 10.3390/toxins8030078 (2016). PMC4810223 26999206

[b20] Escoubas P. . In 15th World Congress on Animal, Plant and Microbial Toxins 220–221 (Glasgow, Scotland, 2006).

[b21] Minett M. S. . Endogenous opioids contribute to insensitivity to pain in humans and mice lacking sodium channel Nav1.7. Nature communications 6, 8967, doi: 10.1038/ncomms9967 (2015). PMC4686868 26634308

[b22] King G. F., Gentz M. C., Escoubas P. & Nicholson G. M. A rational nomenclature for naming peptide toxins from spiders and other venomous animals. Toxicon 52, 264–276, doi: 10.1016/j.toxicon.2008.05.020 (2008). 18619481

[b23] Schmalhofer W. A. . ProTx-II, a selective inhibitor of NaV1.7 sodium channels, blocks action potential propagation in nociceptors. Molecular pharmacology 74, 1476–1484, doi: 10.1124/mol.108.047670 (2008). 18728100

[b24] Alexandrou A. J. . Subtype-Selective Small Molecule Inhibitors Reveal a Fundamental Role for Nav1.7 in Nociceptor Electrogenesis, Axonal Conduction and Presynaptic Release. PLoS One 11, e0152405, doi: 10.1371/journal.pone.0152405 (2016). 27050761 PMC4822888

[b25] Dib-Hajj S. D., Yang Y., Black J. A. & Waxman S. G. The Na(V)1.7 sodium channel: from molecule to man. Nat. Rev. Neurosci. 14, 49–62, doi: 10.1038/nrn3404 (2013). 23232607

[b26] Klint J. K. . Seven novel modulators of the analgesic target NaV 1.7 uncovered using a high-throughput venom-based discovery approach. Br. J. Pharmacol. 172, 2445–2458, doi: 10.1111/bph.13081 (2015). 25754331 PMC4409898

[b27] Bosmans F., Martin-Eauclaire M. F. & Swartz K. J. Deconstructing voltage sensor function and pharmacology in sodium channels. Nature 456, 202–208, doi: 10.1038/nature07473 (2008). 19005548 PMC2587061

[b28] Brünger A. T. . Crystallography & NMR system: A new software suite for macromolecular structure determination. Acta Crystallographica Section D: Biological Crystallography 54, 905–921 (1998). 9757107 10.1107/s0907444998003254

[b29] Takahashi H. . Solution structure of hanatoxin1, a gating modifier of voltage-dependent K^+^ channels: common surface features of gating modifier toxins. Journal of molecular biology 297, 771–780 (2000). 10731427 10.1006/jmbi.2000.3609

[b30] Jung H. J. . Solution structure and lipid membrane partitioning of VSTx1, an inhibitor of the K_v_AP potassium channel. Biochemistry 44, 6015–6023 (2005). 15835890 10.1021/bi0477034

[b31] Minett M. S. . Distinct Nav1.7-dependent pain sensations require different sets of sensory and sympathetic neurons. Nature communications 3, 791, doi: 10.1038/ncomms1795 (2012). PMC3337979 22531176

[b32] Gingras J. . Global Nav1.7 knockout mice recapitulate the phenotype of human congenital indifference to pain. PLoS One 9, e105895, doi: 10.1371/journal.pone.0105895 (2014). 25188265 PMC4154897

[b33] Nassar M. A. . Nociceptor-specific gene deletion reveals a major role for Nav1.7 (PN1) in acute and inflammatory pain. Proceedings of the National Academy of Sciences of the United States of America 101, 12706–12711, doi: 10.1073/pnas.0404915101 (2004). 15314237 PMC515119

[b34] Focken T. . Discovery of Aryl Sulfonamides as Isoform-Selective Inhibitors of NaV1.7 with Efficacy in Rodent Pain Models. ACS Med. Chem. Lett. 7, 277–282, doi: 10.1021/acsmedchemlett.5b00447 (2016). 26985315 PMC4789675

[b35] Sun S., Cohen C. J. & Dehnhardt C. M. Inhibitors of voltage-gated sodium channel Nav1.7: patent applications since 2010. Pharmaceutical patent analyst 3, 509–521, doi: 10.4155/ppa.14.39 (2014). 25374320

[b36] Deuis J. R. . An animal model of oxaliplatin-induced cold allodynia reveals a crucial role for Nav1.6 in peripheral pain pathways. Pain 154, 1749–1757, doi: 10.1016/j.pain.2013.05.032 (2013). 23711479 PMC3748219

[b37] Deuis J. R. . Analgesic effects of clinically used compounds in novel mouse models of polyneuropathy induced by oxaliplatin and cisplatin. Neuro-oncology 16, 1324–1332, doi: 10.1093/neuonc/nou048 (2014). 24714523 PMC4165414

[b38] Tanaka B. S. . A gain-of-function mutation in Nav1.6 in a case of trigeminal neuralgia. Mol Med 22, doi: 10.2119/molmed.2016.00131 (2016). PMC5023517 27496104

[b39] Black J. A., Frezel N., Dib-Hajj S. D. & Waxman S. G. Expression of Nav1.7 in DRG neurons extends from peripheral terminals in the skin to central preterminal branches and terminals in the dorsal horn. Molecular pain 8, 82, doi: 10.1186/1744-8069-8-82 (2012). 23134641 PMC3517774

[b40] Cummins T. R., Howe J. R. & Waxman S. G. Slow closed-state inactivation: a novel mechanism underlying ramp currents in cells expressing the hNE/PN1 sodium channel. J. Neurosci. 18, 9607–9619 (1998). 9822722 10.1523/JNEUROSCI.18-23-09607.1998 PMC6793269

[b41] Rush A. M., Cummins T. R. & Waxman S. G. Multiple sodium channels and their roles in electrogenesis within dorsal root ganglion neurons. The Journal of physiology 579, 1–14, doi: 10.1113/jphysiol.2006.121483 (2007). 17158175 PMC2075388

[b42] Deuis J. R. & Vetter I. The thermal probe test: A novel behavioral assay to quantify thermal paw withdrawal thresholds in mice. Temperature 3, 199–207, doi: 10.1080/23328940.2016.1157668 (2016). PMC4965000 27857950

[b43] Fang X. . Intense isolectin-B4 binding in rat dorsal root ganglion neurons distinguishes C-fiber nociceptors with broad action potentials and high Nav1.9 expression. J. Neurosci. 26, 7281–7292, doi: 10.1523/jneurosci.1072-06.2006 (2006). 16822986 PMC6673936

[b44] Fukuoka T. . Comparative study of the distribution of the alpha-subunits of voltage-gated sodium channels in normal and axotomized rat dorsal root ganglion neurons. The Journal of comparative neurology 510, 188–206, doi: 10.1002/cne.21786 (2008). 18615542

[b45] Fukuoka T. & Noguchi K. Comparative study of voltage-gated sodium channel alpha-subunits in non-overlapping four neuronal populations in the rat dorsal root ganglion. Neurosci. Res. 70, 164–171, doi: 10.1016/j.neures.2011.01.020 (2011). 21303679

[b46] Black J. A. . Spinal sensory neurons express multiple sodium channel alpha-subunit mRNAs. Brain Res. Mol. Brain Res. 43, 117–131 (1996). 9037525 10.1016/s0169-328x(96)00163-5

[b47] Weerasuriya A. & Mizisin A. P. The blood-nerve barrier: structure and functional significance. Methods Mol. Biol. 686, 149–173, doi: 10.1007/978-1-60761-938-3_6 (2011). 21082370

[b48] Herzog R. I., Cummins T. R., Ghassemi F., Dib-Hajj S. D. & Waxman S. G. Distinct repriming and closed-state inactivation kinetics of Nav1.6 and Nav1.7 sodium channels in mouse spinal sensory neurons. The Journal of physiology 551, 741–750, doi: 10.1113/jphysiol.2003.047357 (2003). 12843211 PMC2343279

[b49] Renganathan M., Cummins T. R. & Waxman S. G. Contribution of Na(v)1.8 sodium channels to action potential electrogenesis in DRG neurons. J. Neurophysiol. 86, 629–640 (2001). 11495938 10.1152/jn.2001.86.2.629

[b50] Vasylyev D. V., Han C., Zhao P., Dib-Hajj S. & Waxman S. G. Dynamic-clamp analysis of wild-type human Nav1.7 and erythromelalgia mutant channel L858H. J. Neurophysiol. 111, 1429–1443, doi: 10.1152/jn.00763.2013 (2014). 24401712

[b51] Emery E. C. . Novel SCN9A mutations underlying extreme pain phenotypes: unexpected electrophysiological and clinical phenotype correlations. J. Neurosci. 35, 7674–7681, doi: 10.1523/jneurosci.3935-14.2015 (2015). 25995458 PMC4438121

[b52] Minett M. S. . Pain without nociceptors? Nav1.7-independent pain mechanisms. Cell Rep 6, 301–312, doi: 10.1016/j.celrep.2013.12.033 (2014). 24440715 PMC3969273

[b53] Emery E. C., Luiz A. P. & Wood J. N. Nav1.7 and other voltage-gated sodium channels as drug targets for pain relief. Expert Opin. Ther. Targets 20, 975–983, doi: 10.1517/14728222.2016.1162295 (2016). 26941184 PMC4950419

[b54] Mambretti E. M. . Functional and structural characterization of axonal opioid receptors as targets for analgesia. Molecular pain 12, doi: 10.1177/1744806916628734 (2016). PMC4994859 27030709

[b55] Stein C. & Zollner C. Opioids and sensory nerves. Handb. Exp. Pharmacol. 495–518, doi: 10.1007/978-3-540-79090-7_14 (2009). 19655116

[b56] Cao L. . Pharmacological reversal of a pain phenotype in iPSC-derived sensory neurons and patients with inherited erythromelalgia. Sci Transl Med 8, 335ra356, doi: 10.1126/scitranslmed.aad7653 (2016). 27099175

[b57] Herzig V. & Hodgson W. C. Neurotoxic and insecticidal properties of venom from the Australian theraphosid spider Selenotholus foelschei. Neurotoxicology 29, 471–475, doi: 10.1016/j.neuro.2008.03.002 (2008). 18423874

[b58] Deuis J. R. . Activation of kappa Opioid Receptors in Cutaneous Nerve Endings by Conorphin-1, a Novel Subtype-Selective Conopeptide, Does Not Mediate Peripheral Analgesia. ACS Chem. Neurosci. 6, 1751–1758, doi: 10.1021/acschemneuro.5b00113 (2015). 26225903

[b59] Cummins T. R. . Nav1.3 sodium channels: rapid repriming and slow closed-state inactivation display quantitative differences after expression in a mammalian cell line and in spinal sensory neurons. J. Neurosci. 21, 5952–5961 (2001). 11487618 10.1523/JNEUROSCI.21-16-05952.2001 PMC6763143

[b60] Vetter I. & Lewis R. J. Characterization of endogenous calcium responses in neuronal cell lines. Biochem. Pharmacol. 79, 908–920, doi: 10.1016/j.bcp.2009.10.020 (2010). 19883631

[b61] Deuis J. R. . Development of a muO-Conotoxin Analogue with Improved Lipid Membrane Interactions and Potency for the Analgesic Sodium Channel NaV1.8. J. Biol. Chem. 291, 11829–11842, doi: 10.1074/jbc.M116.721662 (2016). 27026701 PMC4882450

[b62] Güntert P. In Protein NMR Techniques 353–378 (Springer, 2004).

[b63] Shen Y. & Bax A. Protein backbone and sidechain torsion angles predicted from NMR chemical shifts using artificial neural networks. Journal of biomolecular NMR 56, 227–241 (2013). 23728592 10.1007/s10858-013-9741-y PMC3701756

[b64] Andersen N. H. . Extracting information from the temperature gradients of polypeptide NH chemical shifts. 1. The importance of conformational averaging. Journal of the American Chemical Society 119, 8547–8561 (1997).

[b65] Davis I. W. . MolProbity: all-atom contacts and structure validation for proteins and nucleic acids. Nucleic acids research 35, W375–W383 (2007). 17452350 10.1093/nar/gkm216 PMC1933162

[b66] Koradi R., Billeter M. & Wüthrich K. MOLMOL: a program for display and analysis of macromolecular structures. Journal of molecular graphics 14, 51–55 (1996). 8744573 10.1016/0263-7855(96)00009-4

[b67] Murali S. S., Napier I. A., Rycroft B. K. & Christie M. J. Opioid-related (ORL1) receptors are enriched in a subpopulation of sensory neurons and prolonged activation produces no functional loss of surface N-type calcium channels. J. Physiol. 590, 1655–1667, doi: 10.1113/jphysiol.2012.228429 (2012). 22371475 PMC3413501

[b68] Imlach W. L., Bhola R. F., May L. T., Christopoulos A. & Christie M. J. A Positive Allosteric Modulator of the Adenosine A1 Receptor Selectively Inhibits Primary Afferent Synaptic Transmission in a Neuropathic Pain Model. Mol. Pharmacol. 88, 460–468, doi: 10.1124/mol.115.099499 (2015). 26104547

[b69] Chaplan S. R., Bach F. W., Pogrel J. W., Chung J. M. & Yaksh T. L. Quantitative assessment of tactile allodynia in the rat paw. J. Neurosci. Methods 53, 55–63 (1994). 7990513 10.1016/0165-0270(94)90144-9

