# Corrigendum: Pharmacological characterisation of the highly Na_V_1.7 selective spider venom peptide Pn3a

**DOI:** 10.1038/srep46816

**Published:** 2017-05-26

**Authors:** Jennifer R. Deuis, Zoltan Dekan, Joshua S. Wingerd, Jennifer J. Smith, Nehan R. Munasinghe, Rebecca F. Bhola, Wendy L. Imlach, Volker Herzig, David A. Armstrong, K. Johan Rosengren, Frank Bosmans, Stephen G. Waxman, Sulayman D. Dib-Hajj, Pierre Escoubas, Michael S. Minett, Macdonald J. Christie, Glenn F. King, Paul F. Alewood, Richard J. Lewis, John N. Wood, Irina Vetter

Scientific Reports 7: Article number: 4088310.1038/srep40883; published online: 01 20 2017; updated: 05 26 2017

In this Article, Affiliation 6 is incorrectly listed as ‘Venomtech, Sophie-Antipolis, 06560, Valbonne, France’. The correct affiliation is listed below:

VenomeTech, Sophie-Antipolis, 06560, Valbonne, France.

