# Supplementary material for: Pharmacological characterisation of the highly NaV1.7 selective spider venom peptide Pn3a
Source: Sci Rep. 2017 Jan 20;7:40883. doi: 10.1038/srep40883 (PMC5247677; doi:10.1038/srep40883)
Supplement: Supplementary Information [file srep40883-s1.doc]

**Pharmacological characterisation of the highly Na_V_1.7 selective spider venom peptide Pn3a**

Jennifer R. Deuis,^1^ Zoltan Dekan,^1^ Joshua S. Wingerd,^1^ Jennifer J. Smith,^1^ Nehan R. Munasinghe,^2^ Rebecca F. Bhola,^2^ Wendy L. Imlach,^2^ Volker Herzig,^1^ David A. Armstrong,^3^ K. Johan Rosengren,^3^ Frank Bosmans,^4^ Stephen G. Waxman,^5^ Sulayman D. Dib-Hajj,^5^ Pierre Escoubas,^6^ Michael S. Minett,^7^ Macdonald J. Christie,^2^ Glenn F. King,^1^ Paul F. Alewood,^1^ Richard J. Lewis,^1^ John N. Wood,^7^ Irina Vetter^1,8^*

| **Supplementary Table 1:** Energy and structural statistics of the 20 lowest energy structures of μ-TRTX-Pn3a calculated using a simulated annealing procedure with CNS | | | | | |
| --- | --- | --- | --- | --- | --- |
|  | **μ-TRTX-Pn3a** | | | **STDEV** | |
| **Energies:** |  | | |  | |
| Overall (kcal/mol) | -1344 | | | 39.9 | |
| Bonds (kcal/mol) | 22.6 | | | 1.48 | |
| Angles (kcal/mol) | 58.6 | | | 4.08 | |
| Improper (kcal/mol) | 20.6 | | | 2.70 | |
| Dihedral (kcal/mol) | 169 | | | 2.99 | |
| Van Der Wall (kcal/mol) | -154 | | | 6.18 | |
| NOE (kcal/mol) | 0.33 | | | 0.03 | |
| cDih (kcal/mol) | 0.29 | | | 0.19 | |
| Electrostatic | -1461 | | | 45.8 | |
|  |  | | |  | |
| **RMSD:** |  | | |  | |
| Bonds (Å) | 1.28e-2 | | | 4.53e-4 | |
| Angles (°) | 1.375 | | | 0.0436 | |
| Improper (Å) | 1.43 | | | 0.1166 | |
| Dihedral (Å) | 41.3 | | | 0.199 | |
| NOE (Å) | 2.53e-2 | | | 1.15e-3 | |
| cDih (Å) | 0.189 | | | 0.0671 | |
|  |  | | |  | |
| **Pairwise RMSD (Residues 2-31)** | | | |  | |
| Backbone atoms (Å) | | 0.33 | | 0.07 | |
| Heavy atoms (Å) | | 116 | | 0.12 | |
|  | |  | |  | |
| **Experimental Data:**  Distance Restraints:  Intra-residue (i- j=0)  Sequential (i-j = 1)  Medium range (i-j <5)  Long range (i-j ≥ 5) | | 111  160  67  149 | |  | |
| Dihedral restraints | | 43 backbone / 19 side chain | | | |
| Hydrogen bond restraints | | 34 (for 17 hbonds) | | |  |
| NOE violations >0.3 Å | | 0 | |  | |
| cDih violations> 3.0° | | 1 | |  | |
| **Ramachandran statistics** | | | | | |
| Residues in most favoured regions (%) | | | 92 | | |
| Residues in additionally allowed regions (%) | | | 8 | | |
